# WAT-to-BAT communication facilitates the sustained activation of BAT thermogenesis during cold exposure

**DOI:** 10.1038/s41421-026-00891-8

**Published:** 2026-05-26

**Authors:** Jieyuan Xue, Ding Chen, Chenfeng Wang, Jue Wang, Jiayu Zhou, Wenyan Li, Junxi Qian, Yuanji Huang, Liang Fang, Hongyong Song, Ben He, Xu-Yun Zhao

**Affiliations:** 1 Ruijin Hospital Lu Wan Branch, Shanghai Jiao Tong University School of Medicine, Shanghai, China; 2 Department of Biochemistry and Molecular Cell Biology, Shanghai Key Laboratory for Tumor Microenvironment and Inflammation, Key Laboratory of Cell Differentiation and Apoptosis of National Ministry of Education, Shanghai Jiao Tong University School of Medicine, Shanghai, China; 3 Department of Cardiology, Shanghai Chest Hospital, Shanghai Jiao Tong University School of Medicine, Shanghai, China; 4 Department of Neurosurgery, Renji Hospital, Shanghai Jiao Tong University School of Medicine, Shanghai, China; 5 Shanghai East Hospital, Tongji University School of Medicine, Shanghai, China

**Keywords:** Hormone receptors, Extracellular signalling molecules

## Abstract

The activation of brown adipose tissue (BAT) for thermogenesis represents a crucial physiological mechanism that helps maintain body temperature during cold exposure. Nevertheless, the exact mechanisms underlying the sustained activation of BAT under cold conditions remain incompletely understood. In this study, we reveal that soluble ST2 (sST2) mediates a white adipose tissue (WAT)-to-BAT endocrine mechanism that is essential for the continuous activation of BAT during cold exposure. Specific depletion of sST2 blocks alternative thermogenesis following BAT denervation and renders mice sensitive to cold during prolonged cold exposure. Mechanistically, sST2 is induced and secreted from epididymal white adipose tissue (eWAT) upon the activation of Creb1, which is driven by β1 and β2 adrenergic receptor signaling. Secreted sST2 directly binds to the β3 adrenergic receptor in BAT and, in synergy with norepinephrine, induces BAT thermogenesis independent of IL33. Additionally, supplementation with sST2 promotes beige fat formation. Therefore, our study illustrates a novel mechanism through which the adipokine sST2 derived from eWAT mediates sustained BAT activation during cold exposure through the integration of neural and humoral signals. More importantly, sST2 exerts a synergistic effect on BAT activation when combined with β3-adrenergic receptor agonists.

## Introduction

Activation of thermogenic adipose tissue, primarily brown adipose tissue (BAT) and beige adipose tissue, the latter of which originates within white adipose tissue (WAT) in response to cold stimulation, is a critical physiological response during cold exposure, enabling organisms to maintain core body temperature^1,2^. These specialized adipose depots dissipate chemical energy as heat and constitute a central component of non-shivering thermogenesis. Among the known mechanisms, uncoupled mitochondrial respiration mediated by uncoupling protein 1 (UCP1) represents the predominant driver of thermogenic activity in brown and beige adipocytes^3^. In addition to thermoregulation, accumulating evidence indicates that the activation of thermogenic adipose tissue can influence systemic metabolic homeostasis^1^. Although the extent to which BAT activation contributes to metabolic regulation in humans remains an area of active investigation, experimental and clinical studies suggest that enhancing BAT function may exert beneficial effects on glucose and lipid metabolism under certain physiological or pathological conditions^4–6^. Therefore, a more in-depth mechanistic understanding of BAT thermogenic regulation is warranted, as it may inform strategies aimed at correcting energy imbalance and improving metabolic health.

The activation of thermogenesis in adipose tissue is orchestrated by the complex interplay of neural and humoral regulatory mechanisms^7,8^. From a neural perspective, the sympathetic nervous system (SNS) plays a central role in the rapid activation of thermogenesis in adipose tissue^9–11^. The prevailing model posits that in response to cold or other thermogenic stimuli, the central nervous system stimulates sympathetic neurons to release norepinephrine (NE), which binds to adrenergic receptors (ARs) in both mice and humans, especially the β3 adrenergic receptor (ADRB3, β3-AR), which is expressed on mouse brown adipocytes (BACs)^12–16^. NE‒ADRB3 binding initiates a classical intracellular signaling cascade through the activation of adenylate cyclase (AC), an increase in cyclic adenosine monophosphate (cAMP) levels, and the subsequent activation of protein kinase A (PKA)^17^. PKA, in turn, phosphorylates key downstream targets, including the transcription factor cAMP responsive element binding protein 1 (CREB1), which induces the transcription of thermogenic genes such as *Ucp1*^18^. In addition to NE, sympathetic nerve terminals also release neuropeptide Y (NPY), which acts on mural cells in BAT to promote their proliferation and sustain the thermogenic capacity of the tissue^19^. Furthermore, dorsal root ganglia (DRG) are responsible for the sensory innervation of subcutaneous fat, allowing the central nervous system to monitor adipose tissue status and coordinate thermogenic responses^20^. Collectively, these findings highlight an intricate and critically essential neural network that governs adipose thermogenesis.

During cold exposure, BAT and other peripheral organs can release hormones and small molecules to promote thermogenesis in BAT through both local and systemic mechanisms. Adipocytes within BAT and beige fat produce a variety of adipokines, such as Adissp, NRG4, and FSTL1, that contribute to BAC thermogenic activation under cold stimulation^21–23^. As a central metabolic organ, the liver also contributes to thermoregulation by secreting hepatokines, such as FGF21 and BMP9, that directly enhance thermogenesis^24,25^. Other tissues, including the thyroid gland and adrenal gland, also secrete thermogenesis-promoting factors such as thyroid hormone and adrenocorticotropic hormone (ACTH)^26,27^. Although substantial research has focused on endocrine factors that regulate BAT thermogenesis, the molecular mechanisms driving BAT activation during the acute phase of cold exposure, particularly the dynamic integration of neural and humoral regulatory pathways, remain largely uncharacterized. Moreover, the role of WAT, a critical secretory tissue in both humans and mice, in thermogenic regulation during acute cold exposure remains poorly understood.

Thermogenic adipose tissue has been shown to contribute to the maintenance of energy homeostasis and metabolic balance, with extensive metabolic benefits demonstrated in animal models. Specifically, in these models, brown and beige adipose tissues contribute to whole-body energy regulation by improving glucose and lipid metabolism and increasing insulin sensitivity^3,5,28–30^. Although the contribution of fat thermogenesis to systemic metabolism in humans is subject to debate, accumulating evidence from recent experimental and clinical studies supports a generally beneficial role under specific physiological or pathological conditions^2,6,31,32^. Accordingly, stimulation of thermogenic activity in brown or beige adipose tissue has emerged as a potential therapeutic approach for metabolic diseases^33–36^.

Beige adipose tissue arises within WAT in response to chronic cold exposure or sustained β-adrenergic signaling. During this process, a subset of white adipocytes acquire BAC-like features, including increased UCP1 expression and enhanced thermogenic capacity^37^. The ability of mirabegron, a selective ADRB3 agonist, to enhance insulin sensitivity and regulate glucose and lipid metabolism by promoting human fat beiging has been demonstrated in preclinical animal models and clinical trials^38,39^. Consequently, therapeutic strategies aimed at activating beige fat offer significant promise for treating obesity and associated metabolic disorders^29,37,40^.

Soluble ST2 (sST2) is an alternatively spliced isoform of the *Il1rl1* gene. Functionally, it acts as a soluble decoy receptor that inhibits the IL-33/ST2L signaling pathway^41^. Unlike ST2L, which is membrane-bound, sST2 is a secreted protein that circulates throughout the body and can be readily detected in serum and other biological fluids^42–45^. A growing body of research has demonstrated that circulating sST2 levels reflect systemic inflammation, immune activation, and cardiovascular status^46–48^. As a decoy receptor in the IL-33/ST2L signaling axis, sST2 levels significantly increase within minutes following inhalation of an allergen, which is followed by an increase in serum IL-33 levels^49,50^. Recent findings have highlighted the pivotal role of the IL-33/ST2L signaling pathway in maintaining adipose tissue homeostasis and promoting the formation of beige fat^51^. Adipocytes, adipocyte progenitor cells, and mesenchymal stromal cells within beige adipose tissue, as well as sympathetic perineurial barrier cells in BAT, have been identified as primary sources of IL-33^52–56^. These studies also underscore the crucial roles of mesenchymal and immune cells in orchestrating adipose tissue remodeling. However, the source and specific involvement of sST2 in modulating IL-33/ST2L signaling during adipose tissue homeostasis and thermogenesis remain poorly understood and warrant further investigation.

In this study, we revealed that over an extended period of cold exposure, the NE-mediated neural regulation of BAT thermogenesis becomes attenuated. As an alternative compensatory mechanism, a communication pathway between WAT and BAT is activated to ensure the continuous thermogenic function of BAT during prolonged cold stress. This process is mediated by sST2, a secreted factor originating from epididymal white adipose tissue (eWAT). The production of sST2 in eWAT is induced by neural-driven β1/2-adrenergic signaling. Once secreted, sST2 can directly bind to ADRB3 on BACs. This binding event triggers β-adrenergic signaling and enhances the thermogenic capacity of BAT through an IL33-independent mechanism. Thus, sST2 orchestrates neural and humoral mechanisms to drive progressive BAT activation through inter-tissue communication between WAT and BAT. Notably, this regulatory signaling pathway can synergize with ADRB3 agonists to drive the formation of beige adipose tissue. Our findings thereby offer a novel perspective on the activation of thermogenic fat.

## Results

### An alternative humoral mechanism maintains the sustained activation of BAT thermogenesis when NE levels are depleted in BAT during cold exposure

**Fig. 1:**
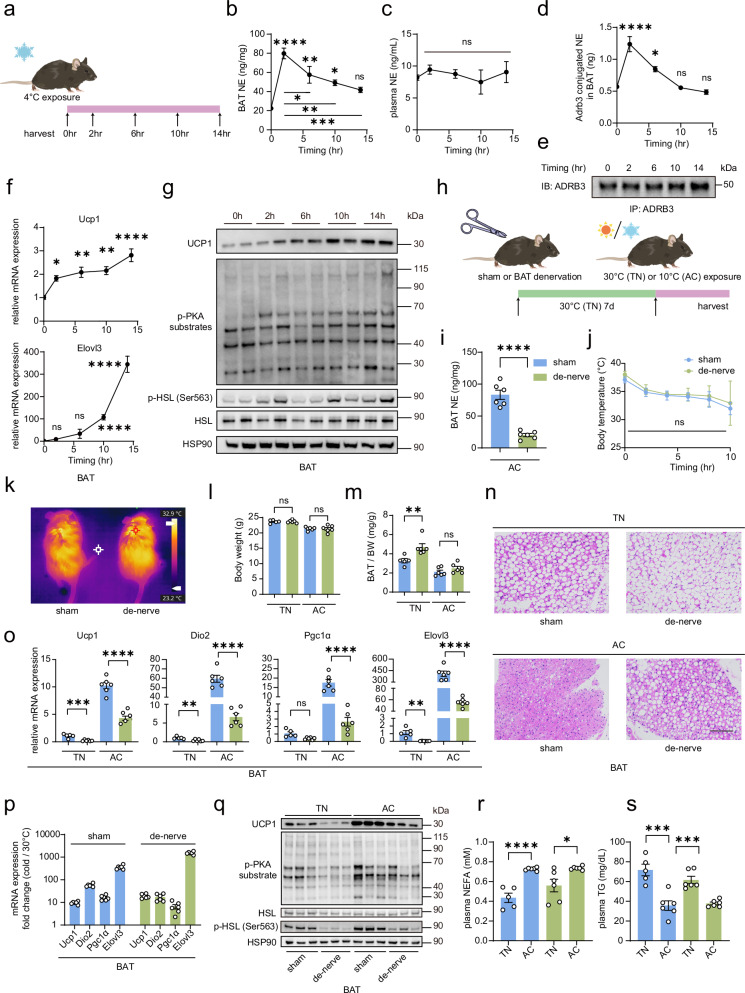
An alternative mechanism of BAT activation is triggered by humoral signaling when NE levels are depleted in BAT during cold exposure. **a** Schematic depicting 10-week-old wild-type (WT) mice randomized into five groups (*n* = 4 per group) and continuously exposed to 4 °C for the indicated durations. **b**–**d** The mice were exposed to 4 °C for the indicated durations (*n* = 4 per group). Line charts showing NE levels in BAT (**b**) plasma (**c**) and ADRB3-immunoprecipitated samples from BAT (**d**). **e** Immunoblotting of ADRB3 immunoprecipitated from BAT in **d**. **f** Line charts showing *Ucp1* and *Elovl3* gene expression by quantitative PCR (qPCR) analysis in BAT from mice exposed to 4 °C at the indicated time points (*n* = 4 per group). **g** Immunoblotting showing the levels of UCP1, phosphorylated PKA substrates, phosphorylated HSL, and total HSL in BAT upon cold exposure at the indicated time points. HSP90 was used as a loading control. **h** Schematic depicting 10-week-old WT mice randomized into sham-operated and BAT-denervated (de-nerve) groups, both of which were exposed to 30 °C (thermoneutrality, TN) or 10 °C (acute cold, AC) for 8 h. **i** NE levels in BAT from sham-operated or BAT-denervated (*n* = 6 per group) mice exposed to cold stimulus at 10 °C (AC). **j** Line chart showing the core body temperature of sham-operated or BAT-denervated (*n* = 6 per group) mice exposed to 10 °C at the indicated time points. **k** Representative infrared thermography image of sham-operated or BAT-denervated mice exposed to 10 °C for 8 h. **l**, **m** Body weight (**l**) and BAT weight-to-body weight (BW) ratio (**m**) of sham-operated or BAT-denervated mice exposed to TN and AC (*n* = 5–6 per group). **n** H&E staining of BAT sections from sham-operated or BAT-denervated (de-nerve) mice exposed to TN and AC. Scale bar, 100 μm. **o** qPCR analysis of thermogenic gene expression in BAT from sham-operated or BAT-denervated mice exposed to TN and AC (*n* = 5–6 per group). **p** Fold change in thermogenic gene mRNA expression in **o** (*n* = 6 per group). **q** Immunoblotting showing the levels of UCP1, phosphorylated PKA substrate, phosphorylated HSL, and total HSL in BAT from sham-operated or BAT-denervated mice exposed to TN and AC. HSP90 was used as a loading control. **r**, **s** Plasma NEFA (**r**) and TG (**s**) levels in sham-operated or BAT-denervated mice exposed to TN and AC (*n* = 5–6 per group). Data are shown as mean ± SEM. Statistical significance was determined by two-tailed unpaired Student’s *t*-test in **i**, **l**, **m**, **o**, **r**, **s**, and one-way ANOVA with multiple comparisons tests in **b**–**d**, **f**, **j**. *P < 0.05, **P < 0.01, ***P < 0.001, ****P < 0.0001; ns not significant.

To comprehensively investigate the dynamics of BAT activation during cold exposure, we exposed a group of mice to a 4 °C environment for a duration of time (Fig. 1a). During cold exposure, the SNS promptly releases NE at neuronal synapses within BAT. NE subsequently binds to and activates ADRB3 (β3-AR) to stimulate thermogenesis^13^. As expected, we detected a transient increase in NE levels in BAT within the first 2 h of cold exposure. However, this was followed by a marked decline over time, while the NE levels in the plasma remained stable (Fig. 1b, c). Moreover, the amount of NE bound to ADRB3 tended to be similar to that of NE in BAT. These findings indicate that the decrease in NE levels within BAT also results in a reduction in NE binding to β-AR (Fig. 1d, e). Intriguingly, despite the substantial decrease in NE levels in BAT, the core body temperature of the mice was maintained (Supplementary Fig. S1a). Histological analysis revealed that the degree of BAT but not eWAT browning continuously increased throughout the entire duration of cold exposure (Supplementary Fig. S1b). Notably, the expression of key thermogenic genes, such as *Ucp1* and *Elovl3*, was persistently upregulated during cold exposure (Fig. 1f, g). Western blot analysis revealed that the expression of β-adrenergic signaling markers, phosphorylated PKA substrates and hormone-sensitive lipase (HSL), which are involved in BAT activation, initially peaked at 2 h of cold exposure along with the elevated NE level. After the level of NE decreased, the levels of phosphorylated PKA substrates and HSL continued to increase and were not correlated with the decrease in NE levels (Fig. 1g). Additionally, we observed an overall increase in non-esterified fatty acid (NEFA) levels and a decrease in triglyceride (TG) levels in plasma, although the NEFA level also peaked at 2 h (Supplementary Fig. S1c, d). These findings suggest that active lipolysis, enhanced fat mobilization, and efficient fatty acid utilization occur continuously during cold exposure for heat production by BAT, even when NE levels and its binding to ADRB3 are reduced. These findings prompted us to hypothesize that NE-independent mechanisms in BAT contribute to the sustained activation of BAT thermogenesis during prolonged cold exposure.

To explore an alternative pathway in BAT activation, we surgically resected BAT sympathetic nerves from mice, effectively eliminating NE within the tissue (Fig. 1h, i). During the first 4 h of cold exposure at 4 °C, these BAT-denervated mice exhibited significant hypothermia. This clearly indicates that the initial stimulation of BAT by NE is essential for rapid thermogenesis during cold exposure (Supplementary Fig. S1e, f). To investigate the NE-independent mechanisms involved in BAT thermogenesis, we subjected BAT-denervated mice to a mildly cold environment (10 °C) (Fig. 1h). Notably, compared with the sham-operated mice, the BAT-denervated mice were able to maintain their body temperature during 10 °C cold exposure, with no significant difference in BAT thermogenesis (Fig. 1j, k). Notably, although BAT denervation did not affect body weight, it substantially increased BAT weight, and this effect was alleviated after cold exposure (Fig. 1l, m). Hematoxylin‒eosin (H&E) staining revealed an increased number of lipid droplets in the BAT of denervated mice. Interestingly, despite this initial increase, the number of these lipid droplets decreased upon cold exposure, suggesting that the lipolysis activity of BAT was maintained in BAT-denervated mice (Fig. 1n). Furthermore, although the expression of thermogenic genes, including *Ucp1*, *Dio2*, *Pgc1α*, and *Elovl3*, as well as that of β-adrenergic signaling indicators, phosphorylated PKA substrates and HSL, was largely inhibited in BAT-denervated mice, these factors were strongly induced upon cold exposure (Fig. 1o–q). In addition, plasma glucose and TG levels decreased, whereas NEFA levels increased, suggesting enhanced glucose uptake and lipolysis in adipose tissue during cold exposure in BAT-denervated mice (Fig. 1r, s; Supplementary Fig. S1g). Collectively, these findings offer compelling evidence that an NE-independent pathway in BAT is activated in BAT-denervated mice to drive BAT thermogenesis in response to cold exposure.

### Cold induces sST2 expression and secretion in white adipocytes to enhance β-adrenergic signaling in BAT

**Fig. 2:**
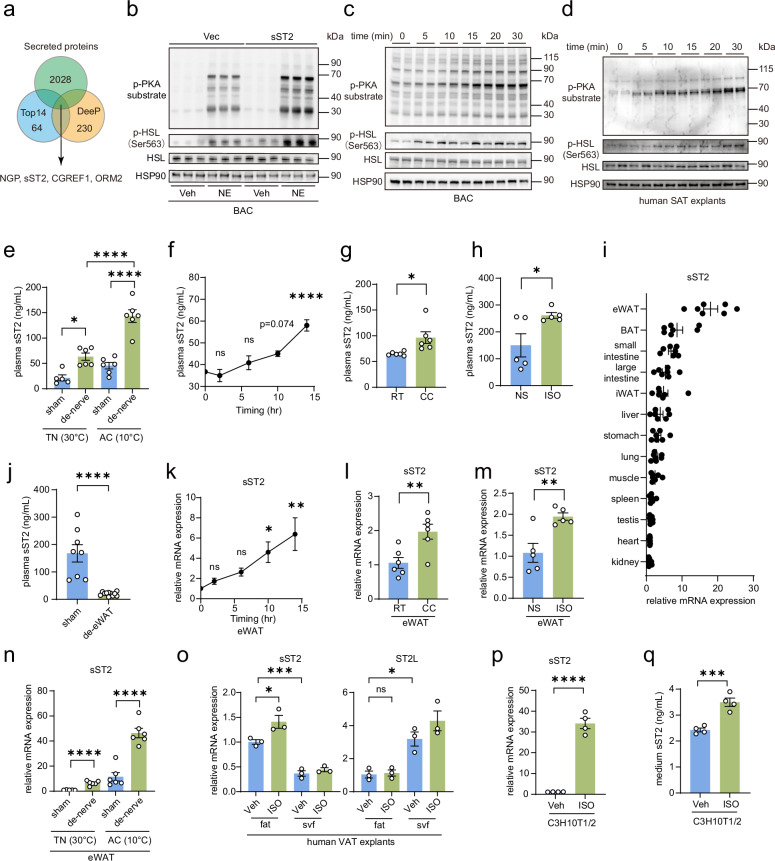
Cold induces the expression and secretion of sST2 in white adipocytes, thereby enhancing β-adrenergic signaling in BAT. **a** Venn diagram comparing the plasma proteomic profiles of mice with BAT denervation under cold exposure (10 °C) versus thermoneutral conditions (30 °C). Proteomic analysis was performed using two high-abundance protein depletion methods: High Select™ Top14 Abundant Protein Depletion Mini Spin Columns (Top14) and EasyPept DeeP (DeeP). Proteins enriched under cold conditions were annotated against known secreted protein databases. **b** Immunoblotting showing the levels of phosphorylation of the PKA substrate and HSL in vector (Vec)- and sST2-overexpressing BACs after differentiation, following treatment with vehicle (Veh) or NE (1 µM) for 15 min. HSP90 was used as a loading control. **c**, **d** Immunoblotting showing phosphorylation of the PKA substrate and HSL in differentiated BACs (**c**) and human subcutaneous adipose tissue (SAT) explants (**d**) stimulated with 5 µg/mL recombinant sST2 protein for the indicated durations. HSP90 was used as a loading control. **e** Plasma sST2 levels in sham-operated or BAT-denervated mice exposed to 30 °C (thermoneutrality, TN) or 10 °C (acute cold, AC) (*n* = 5–6 per group). **f** Line chart showing plasma sST2 levels in mice (*n* = 4 per group) exposed to 4 °C at the indicated time points. **g** Plasma sST2 levels in mice (*n* = 6 per group) exposed to chronic cold (CC) or at room temperature (RT) for 7 days. **h** Plasma sST2 levels in mice (*n* = 5 per group) that were intraperitoneally injected with saline (NS) or ISO (30 mg/kg/d) for 7 days. **i** qPCR analysis of sST2 expression in a panel of tissues from 10-week-old WT mice (*n* = 7 per group). **j** Plasma sST2 levels in mice after sham surgery (*n* = 8) or after eWAT removal (de-eWAT, *n* = 11). **k** Line chart showing sST2 expression by qPCR analysis in eWAT from mice exposed to 4 °C at the indicated time points (*n* = 4 per group). **l** qPCR analysis of sST2 expression in eWAT from mice (*n* = 6 per group) exposed to CC or at RT for 7 days. **m** qPCR analysis of sST2 expression in eWAT from mice (*n* = 5 per group) that were intraperitoneally injected with saline (NS) or ISO (30 mg/kg/d) for 7 days. **n** qPCR analysis of sST2 expression in eWAT from mice subjected to sham surgery or BAT denervation and exposed to TN or AC (*n* = 5–6 per group). **o** qPCR analysis of sST2 and ST2L expression in adipocytes (fat) and SVF isolated from human VAT explants (*n* = 3 per group) treated with vehicle (Veh) or ISO (1 µM) for 24 h. **p**, **q** qPCR analysis of sST2 expression (**p**) in differentiated C3H10T1/2 cells (*n* = 4 per group) and quantification of secreted sST2 levels in culture medium (**q**) after treatment with vehicle (Veh) or ISO (1 µM) for 24 h. Data are shown as mean ± SEM. Statistical significance was determined by two-tailed unpaired Student’s *t*-test in **g**, **h**, **j**, **l**–**n**, **p**, **q**, and one-way ANOVA with multiple comparisons tests in **e**, **f**, **k**, **o**. *P < 0.05, **P < 0.01, ***P < 0.001, ****P < 0.0001; ns not significant.

Secreted factors play pivotal roles as regulators of BAT thermogenesis^57,58^. We postulate that the alternative activation of BAT through β-adrenergic signaling, independent of NE, is mediated by a cold-induced secreted factor during cold exposure. Given that fibroblast growth factor 21 (Fgf21), a well-known hepatokine, promotes BAT thermogenesis in response to chronic cold, we initially investigated the expression of Fgf21 in the livers of BAT-denervated mice following cold exposure^24^. However, Fgf21 expression was not altered in these mice (Supplementary Fig. S2a). To unbiasedly identify the secreted factor that directly activates β-adrenergic signaling for thermogenesis during cold exposure in BAT-denervated mice, we subjected the plasma from both sham-operated and BAT-denervated mice after cold exposure to mass spectrometry analysis. By employing two independent peptide purification methods (column-based and magnetic bead-based), we identified four secreted factors, namely, neutrophilic granule protein (Ngp), sST2, cell growth regulator with EF hand domain protein 1 (Cgref1), and orosomucoid 2 (Orm2), whose expression was significantly upregulated in BAT-denervated mice (Fig. 2a). To confirm the effects of these secreted factors on the β-adrenergic activation of BAT, we individually overexpressed each factor in primary brown preadipocytes (Supplementary Fig. S2b). After their differentiation into mature BACs, the levels of phosphorylated PKA substrates and HSL were evaluated to assess β-adrenergic signaling activation. We found that only sST2 overexpression led to an increase in the phosphorylation of PKA substrates and HSL upon NE treatment (Fig. 2b; Supplementary Fig. S2c). Additionally, treatment with recombinant sST2 directly activated β-adrenergic signaling, as demonstrated by enhanced phosphorylation of PKA substrates and HSL in both murine BACs after differentiation and human subcutaneous fat explants (Fig. 2c, d). Previous studies have shown that lipolysis induces the expression of Gpr3 in BAT and that its endogenous ligand, oleic acid, promotes thermogenesis under cold exposure^59,60^. However, in our study, we found that the expression of Gpr3 in BAT and BACs was markedly lower than that of the three β-AR isoforms (Supplementary Fig. S2d, e). To investigate whether oleic acid could activate PKA signaling, we treated differentiated BACs with oleic acid in vitro. The results revealed no significant changes in PKA substrate phosphorylation upon oleic acid treatment, suggesting that oleic acid is unlikely to activate Gpr3-mediated PKA activation, probably due to the very low expression of Gpr3 (Supplementary Fig. S2f). These results suggest that the secretion of sST2 in BAT-denervated mice may represent the primary mechanism mediating alternative BAT activation in the absence of local NE by directly triggering β-adrenergic signaling. Moreover, this effect appears to act synergistically with NE in inducing thermogenesis.

Following up on this notion, we confirmed that the plasma sST2 levels in BAT-denervated mice were elevated, with the levels of sST2 further increasing after cold exposure (Fig. 2e). Notably, plasma sST2 levels were consistently upregulated over time during 4 °C cold exposure (Fig. 2f). Additionally, sST2 secretion was elevated in mice after chronic cold exposure, as well as after treatment with isoprenaline (ISO), a non-selective β-AR agonist, for 7 days (Fig. 2g, h). These findings indicate that sST2 expression is induced by cold and may play a role in activating β-adrenergic signaling in BAT.

Our previous research convincingly demonstrated that sST2 functions as an adipokine that regulates the homeostasis of regulatory T cells (Tregs) and Group 2 innate lymphoid cells (ILC2s) within adipose tissue^61^. Concordantly, among various tissues, the expression of sST2 was the highest in eWAT (Fig. 2i). Significantly, following the surgical excision of eWAT from mice, plasma sST2 levels markedly decreased (Fig. 2j). These findings corroborate the conclusion that sST2 is an adipokine originating from eWAT.

To explore whether the increase in circulating sST2 levels during cold exposure is attributed to the upregulation of sST2 expression in eWAT rather than BAT, we measured the expression of sST2 in eWAT and BAT at different time points following cold exposure. In line with the levels of plasma sST2, cold exposure induced a time-dependent increase in the expression of sST2 within eWAT but not BAT (Fig. 2k; Supplementary Fig. S2g). Furthermore, both chronic cold exposure and ISO treatment significantly augmented the expression of sST2 in eWAT but not BAT, suggesting that cold-induced β-adrenergic signaling in eWAT drives sST2 expression (Fig. 2l, m; Supplementary Fig. S2h, i). Interestingly, the denervation of BAT significantly elevated sST2 expression in eWAT, suggesting that compensatory activation of β-adrenergic signaling may occur in eWAT and subsequently induce sST2 expression. This phenotype was not observed in BAT (Fig. 2n; Supplementary Fig. S2j).

Our previous study demonstrated that sST2 is predominantly expressed in mature adipocytes rather than in the stromal vascular fraction (SVF)^61^. To determine whether cold exposure induces sST2 expression in eWAT adipocytes, we isolated adipocytes and SVFs from clinical visceral fat samples. Consistent with our findings in mice, sST2 expression was higher in human adipocytes than in SVFs, and was upregulated by ISO treatment (Fig. 2o). Moreover, treatment with ISO directly led to the significant upregulation of sST2 expression in the white adipocyte cell line C3H10T1/2 after differentiation. Furthermore, the levels of secreted sST2 increased in the culture medium (Fig. 2p, q). These findings demonstrate that cold exposure elicits the expression of sST2 in eWAT adipocytes. This, in turn, results in an augmented secretion of sST2 from eWAT. Given that sST2 directly stimulates β-adrenergic signaling within BAT, these results suggest the establishment of an sST2-mediated regulatory axis between white and brown fat, which may serve as an alternative mechanism to activate BAT thermogenesis during cold exposure.

### ADRB1- and ADRB2-mediated β-adrenergic signaling induces sST2 expression in eWAT

**Fig. 3:**
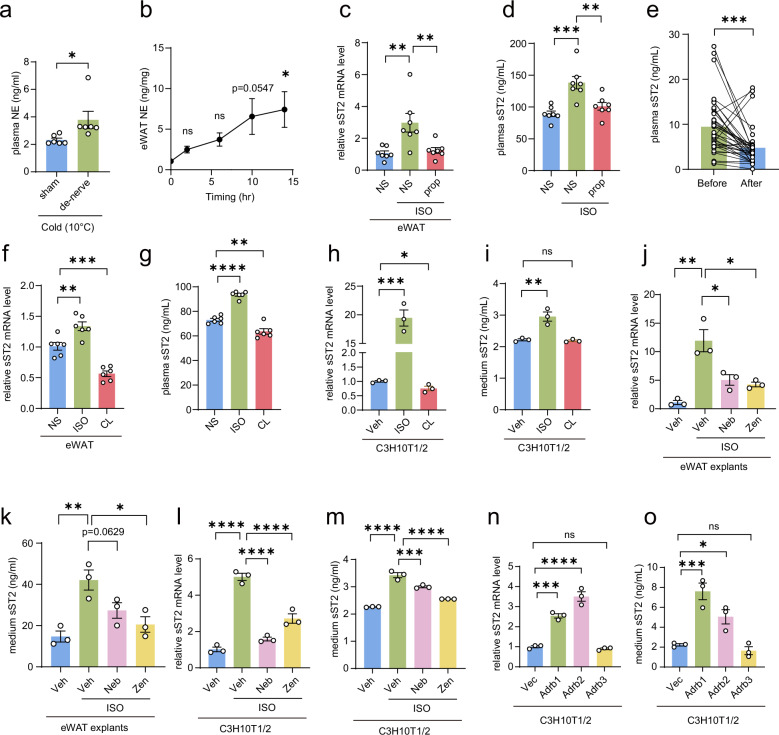
β-Adrenergic signaling mediated by ADRB1 and ADRB2 induces sST2 expression in eWAT. **a** Plasma NE levels in mice subjected to sham surgery or BAT denervation (*n* = 6 per group) and exposed to cold stimulus at 10 °C (acute cold, AC). **b** Line chart showing NE levels in eWAT from mice (*n* = 4 per group) exposed to 4 °C at the indicated time points. **c**, **d** qPCR analysis of sST2 expression in eWAT (**c**) and plasma sST2 levels (**d**) from mice intraperitoneally injected with saline (NS) or a combination of ISO (30 mg/kg/d) and NS or propranolol (prop, 30 mg/kg/d) for 14 days (*n* = 7 per group). **e** Plasma sST2 levels in clinical patients before and after β-AR blocker (β-blocker) administration (*n* = 37 paired samples). **f**, **g** qPCR analysis of sST2 expression in eWAT (**f**) and plasma sST2 levels (**g**) from mice intraperitoneally injected with NS, ISO (100 mg/kg), or CL 316,243 (CL, 1 mg/kg) for 24 h (*n* = 6 per group). **h**, **i** qPCR analysis of sST2 expression in differentiated C3H10T1/2 cells (**h**) and quantification of secreted sST2 levels in culture medium (**i**) after treatment with vehicle (Veh), ISO (1 µM), or CL (1 µM) for 24 h (*n* = 3 per group). **j**, **k** qPCR analysis of sST2 expression in in vitro-cultured eWAT explants from WT mice (**j**) and quantification of secreted sST2 levels in the culture medium (**k**) after treatment with Veh or a combination of ISO (1 µM) and Veh or nebivolol (Neb, 1 µM) or zenidolol (Zen, 1 µM) (*n* = 3 per group). **l**, **m** qPCR analysis of sST2 expression in differentiated C3H10T1/2 cells (**l**) and quantification of secreted sST2 levels in culture medium (**m**) after treatment with Veh or a combination of ISO (1 µM) and Veh, Neb (1 µM) or Zen (1 µM) (*n* = 3 per group). **n**, **o** qPCR analysis of sST2 expression in differentiated C3H10T1/2 cells (**n**) and quantification of secreted sST2 levels in culture medium (**o**) following overexpression of vector (Vec), Adrb1, Adrb2, or Adrb3 (*n* = 3 per group). Data are shown as mean ± SEM. Statistical significance was determined by two-tailed unpaired Student’s *t*-test in **a**, two-tailed paired Student’s *t*-test in **e**, and one-way ANOVA with multiple comparisons tests in **b**–**d**, **f**–**o**. *P < 0.05, **P < 0.01, ***P < 0.001, ****P < 0.0001; ns not significant.

NE is the primary catecholamine released by the SNS to activate β-adrenergic signaling upon cold exposure^13^. We confirmed that plasma NE levels were elevated in BAT-denervated mice, which may contribute to the induction of sST2 expression and secretion in these mice (Fig. 3a). More importantly, the progressive increase in NE levels within eWAT over time during cold exposure was concurrent with the induction of sST2 expression (Fig. 3b). Based on these observations, we hypothesize that sST2 is induced by NE-driven β-adrenergic activation. To test this hypothesis, we treated mice with propranolol, a non-selective β-AR antagonist, in combination with ISO. The results revealed that propranolol treatment significantly suppressed ISO-induced sST2 expression in eWAT, as well as its secretion into the plasma (Fig. 3c, d). Remarkably, in a cohort of cardiovascular disease patients, following treatment with β-AR antagonists, there was an obvious decrease in plasma sST2 levels (Fig. 3e). These findings demonstrate that β-adrenergic signaling elicits the expression and secretion of sST2 in eWAT.

NE activates β-adrenergic signaling by binding to β-AR. In adipose tissue, β-AR exists as three isoforms: ADRB1, ADRB2, and ADRB3. Among them, Adrb3 is the most highly expressed isoform in eWAT and mature C3H10T1/2 adipocytes (Supplementary Fig. S3a). Therefore, we first examined whether Adrb3 is the primary β-AR that mediates sST2 expression in eWAT. Surprisingly, after treatment with CL316,243, a selective ADRB3 agonist, both the plasma sST2 level and the mRNA expression level of sST2 in eWAT decreased, whereas ISO treatment consistently increased sST2 expression and secretion (Fig. 3f, g). This differential effect of ISO and CL316,243 on sST2 expression and secretion was replicated in C3H10T1/2 adipocytes treated with each agent individually (Fig. 3h, i). These results suggest that ADRB3 is not the primary β-AR isoform in adipocytes that promotes sST2 expression.

To explore the roles of the other β-AR isoforms, ADRB1 and ADRB2, in ISO-induced sST2 expression in adipose tissue, we treated fat explants from eWAT and differentiated C3H10T1/2 cells with ISO, together with nebivolol and zenidolol, which are selective inhibitors of ADRB1 and ADRB2, respectively. ISO treatment alone significantly increased sST2 expression and secretion in fat explants and mature white adipocytes. However, treatment with nebivolol or zenidolol in combination with ISO partially reversed the upregulation of sST2 expression induced by ISO (Fig. 3j–m). These results indicate that the activation of β1/2-AR-mediated β-adrenergic signaling promotes sST2 expression. To clarify the effects of the three β-AR isoforms on sST2 induction, we overexpressed each of the three receptors in C3H10T1/2 cells (Supplementary Fig. S3b). In line with prior findings, overexpression of Adrb1 and Adrb2 in C3H10T1/2 cells substantially increased both sST2 expression and secretion. In contrast, overexpression of Adrb3 did not affect sST2 induction (Fig. 3n, o). These results demonstrate that sST2 expression in eWAT is selectively induced by ADRB1- and ADRB2-mediated β-adrenergic signaling.

### Creb1 transcriptionally induces sST2 expression and secretion in adipocytes

**Fig. 4:**
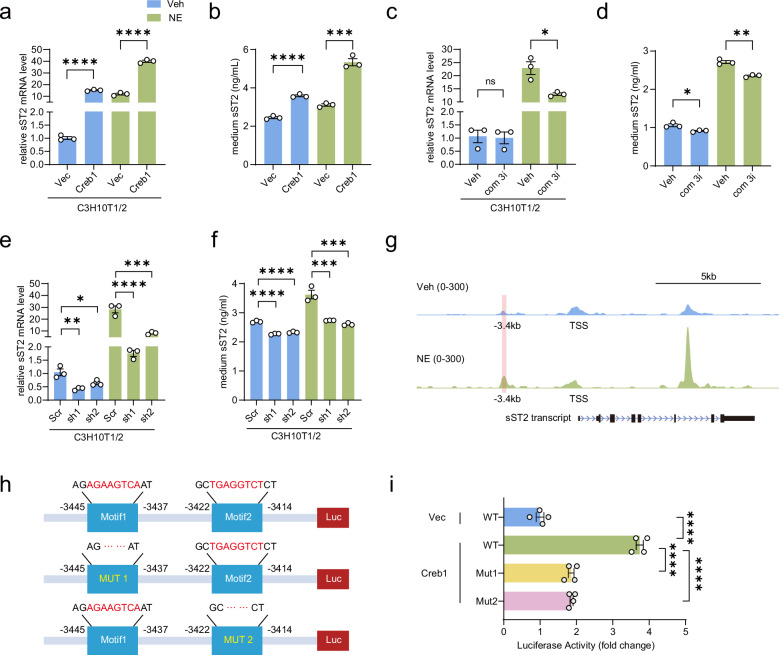
Creb1 drives sST2 transcription and secretion in adipocytes. **a**, **b** qPCR analysis of sST2 expression in differentiated C3H10T1/2 cells (**a**) and quantification of secreted sST2 levels in the culture medium (**b**) after transfection with the vector (Vec) or Creb1-overexpressing plasmid (Creb1), followed by treatment with vehicle (Veh) or NE (1 μM) for 6 h (*n* = 3 per group). **c**, **d** qPCR analysis of sST2 expression in differentiated C3H10T1/2 cells (**c**) and quantification of secreted sST2 levels in the culture medium (**d**) after treatment with Veh or Compound 3i (com 3i) for 24 h, followed by treatment with Veh or NE (1 µM) for 6 h (*n* = 3 per group). **e**, **f** qPCR analysis of sST2 expression in C3H10T1/2 cells (**e**) and quantification of secreted sST2 levels in the culture medium (**f**) after transfection with scrambled (Scr) or two independent *Creb1*-targeting small hairpin RNAs (sh1 and sh2), followed by treatment with Veh or NE (1 µM) for 6 h (*n* = 3 per group). **g** Genome browser visualization of CREB1 occupancy at the sST2 promoter following treatment with Veh or NE (1 μM) for 4 h, as determined by the CUT&Tag assay. Red box: predicted CREB1 binding motif. TSS: transcription start site. **h** Schematic depicting the two Creb1 binding motifs predicted by JASPAR and their respective deletions: Motif 1: AGAAGTCA (positions –3445 bp to –3437 bp); Motif 2: TGAGGTCT (positions –3422 bp to –3414 bp). **i** Luciferase activity of the sST2 promoter measured in HEK293T cells co-transfected with vector (Vec) or pcDNA3-CREB1 (Creb1) and WT or mutant (Mut1 and Mut2) sST2 promoter luciferase plasmids after 24 h of transfection (*n* = 4 per group). Data are shown as mean ± SEM. Statistical significance was determined by two-tailed unpaired Student’s *t*-test in **a**–**d** and one-way ANOVA for multiple comparisons in **e**, **f**, **i**. *P < 0.05, **P < 0.01, ***P < 0.001, ****P < 0.0001; ns not significant.

Creb1 functions as a crucial transcription factor activated by β-adrenergic signaling. Activation of β-AR prompts CREB phosphorylation, enabling CREB to mediate downstream gene expression^62^. To investigate whether β-adrenergic activation-induced sST2 expression is mediated by Creb1, we overexpressed Creb1 in C3H10T1/2 cells and assessed sST2 expression (Supplementary Fig. S4a). The overexpression of Creb1 significantly increased sST2 at the transcriptional level, and this effect was further enhanced upon NE treatment. Consequently, the level of sST2 secreted by Creb1-overexpressing C3H10T1/2 cells also notably increased (Fig. 4a, b). Additionally, to investigate the role of Creb1 in regulating sST2 expression, we used Compound 3i, a specific CREB1 inhibitor^63^. Creb1 is a transcription factor known to promote *Ucp1* transcription. In this study, we confirmed that Compound 3i significantly attenuated the Creb1-mediated activation of the *Ucp1* promoter (Supplementary Fig. S4b)^64^. Notably, treating C3H10T1/2 cells with Compound 3i after NE treatment decreased sST2 expression. Additionally, the amount of sST2 secreted into the culture medium was also reduced (Fig. 4c, d). To validate that Creb1 serves as the key regulator of sST2 in white adipocytes, we knocked down *Creb1* in C3H10T1/2 cells (Supplementary Fig. S4c). Knockdown of *Creb1* resulted in a significant reduction in both basal and NE-stimulated sST2 expression, as well as a decrease in sST2 secretion into the culture medium, confirming that Creb1 is the key transcription factor regulating sST2 expression upon the activation of β-adrenergic signaling (Fig. 4e, f).

To determine whether Creb1 binds directly to the sST2 promoter, we performed Cleavage Under Targets & Tagmentation (CUT&Tag) assays using an antibody against phosphorylated CREB1 on differentiated C3H10T1/2 adipocytes that were treated with vehicle or NE. Our analysis revealed significant enrichment of CREB1 binding within the region spanning –3.45 kb to –3.40 kb upstream of the sST2 transcription start site. Notably, NE treatment further augmented this binding, highlighting its regulatory significance (Fig. 4g). Subsequent luciferase assays with the –4949 bp to +974 bp sST2 promoter region confirmed that Creb1 overexpression markedly boosted luciferase activity, corroborating the hypothesis that CREB1 binds to this region and induces sST2 transcription (Fig. 4h, i). Subsequently, the JASPAR database was utilized to predict two potential Creb1 binding sites in the –3445 bp to –3437 bp and –3422 bp to –3414 bp regions of the sST2 promoter, which is consistent with the CUT&Tag results^65^. Next, site-directed mutagenesis of these predicted binding sites was carried out, and the results demonstrated significant reductions in the luciferase activity of these mutants. This strongly indicates that CREB1 binds to the –3445 bp to –3437 bp and –3422 bp to –3414 bp regions, thereby transcriptionally regulating sST2 expression (Fig. 4h, i).

Taken together, these results indicate that the upregulation of sST2 in white adipocytes is mediated through the engagement of β-adrenergic signaling triggered by ADRB1 and ADRB2, followed by the phosphorylation of CREB1, which transcriptionally induces sST2 expression.

### sST2 deficiency impairs the sustained activation of BAT upon cold exposure

**Fig. 5:**
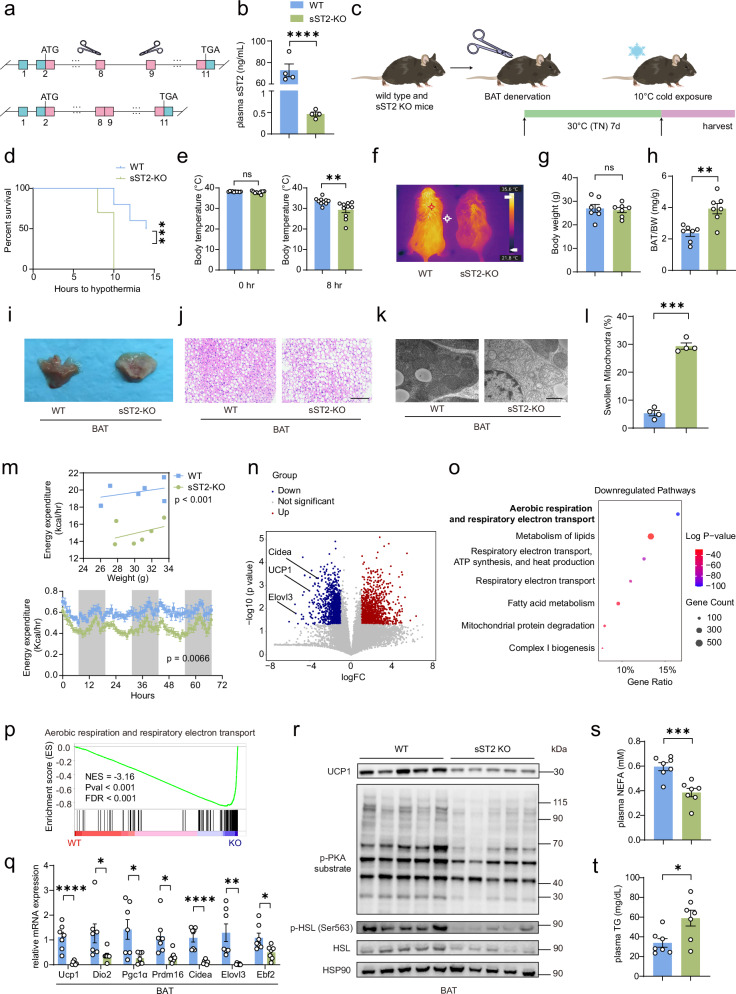
Loss of sST2 impairs the sustained activation of BAT during cold exposure. **a** Schematic depicting the generation of sST2-KO mice using the CRISPR/Cas9 system, leading to the deletion of introns 8–9 (containing the stop codon of sST2) in the *Il1rl1* gene. **b** Plasma sST2 levels in 10-week-old sST2-KO mice and their WT littermates (*n* = 4 per group). **c** Schematic depicting 10-week-old WT and sST2-KO mice subjected to BAT denervation and exposed to cold stimulus at 10 °C for 14 h. **d**, **e** Survival curves (**d**) of WT and sST2-KO mice (*n* = 10 per group) subjected to BAT denervation and exposed to cold stimulus at 10 °C for 14 h. The core body temperature was recorded after 8 h of cold exposure (**e**). The mice were euthanized when their core temperature dropped below 30 °C. **f** Representative infrared thermography images of WT and sST2-KO mice subjected to BAT denervation and exposed to cold stimulus at 10 °C for 8 h. **g**, **h** Body weights (**g**) and BAT weight-to-BW ratios (**h**) of WT and sST2-KO mice (*n* = 7 per group) subjected to BAT denervation and exposed to cold stimulus at 10 °C for 8 h. **i**–**k** Gross morphology (**i**) H&E staining (**j**) and electron microscopy (EM) image (**k**) of BAT from WT and sST2-KO mice after BAT denervation and exposure to cold stimulus at 10 °C for 8 h. Scale bars, 100 μm for H&E staining, 1 μm for EM. **l** Quantification of the percentage of mitochondria with swollen and broken cristae in the BAT EM images (*n* = 4 per group). **m** Regression plots (top) illustrate the trend of increase of energy expenditure with body mass, while line plots (bottom) depict the average energy expenditure recorded over a 68-h period in WT and sST2-KO mice (*n* = 6 per group) following BAT denervation. All measurements were performed in metabolic cages under ambient room temperature conditions. **n** Volcano plot of differentially expressed genes in BAT from sST2-KO mice (*n* = 3 per group) subjected to BAT denervation and exposed to cold stimulus at 10 °C for 8 h compared with WT littermates. Dots highlighted in red and blue represent significantly upregulated (red) and downregulated (blue) genes in BAT from sST2-KO mice. **o** Bubble chart displaying the top 7 downregulated pathways in the Reactome gene sets identified by Metascape enrichment analysis of differentially expressed genes in BAT from sST2-KO mice. **p** GSEA of the indicated pathways in BAT from WT and sST2-KO mice after BAT denervation and exposure to cold stimulus at 10 °C for 8 h. NES: normalized enrichment score; FDR: false discovery rate. **q** qPCR analysis of the expression of thermogenic genes in BAT from WT and sST2-KO mice (*n* = 7 per group) subjected to BAT denervation and exposed to cold stimulus at 10 °C for 8 h. **r** Immunoblotting analyses revealed the levels of UCP1, phosphorylated PKA substrate, phosphorylated HSL, and total HSL in BAT from WT and sST2 KO mice after BAT denervation and exposure to cold stimulus at 10 °C for 8 h. HSP90 was used as a loading control. **s**, **t** NEFA (**s**) and TG (**t**) levels in plasma from WT and sST2-KO mice (*n* = 7 per group) after BAT denervation and exposure to cold stimulus at 10 °C for 8 h. Data are shown as mean ± SEM. Statistical significance was determined by two-tailed unpaired Student’s *t*-test in **b**, **e**, **g**, **h**, **l**, **q**, **s**, **t**; log-rank (Mantel–Cox) tests in **d**; ANCOVA (**m**, top); and two-way ANOVA (**m**, bottom). *P < 0.05, **P < 0.01, ***P < 0.001, ****P < 0.0001; ns not significant.

Our previous results suggested that the depletion of NE in BAT may alternatively activate BAT thermogenesis through the induction of sST2 expression in eWAT. To investigate the role of sST2 in BAT activation during cold exposure upon BAT denervation, we generated an sST2-knockout (KO) mouse model by removing the intron between exons 8 and 9 of the *Il1rl1* gene. This modification abolished the normal termination of the translation of the sST2 transcript, thus specifically depleting the soluble isoform sST2 while maintaining the normal expression of the receptor isoform ST2L (Fig. 5a). As expected, sST2-KO mice exhibited a significant decrease in plasma sST2 levels (Fig. 5b), and the expression of sST2 in eWAT substantially decreased, whereas the expression of ST2L remained unchanged (Supplementary Fig. S5a).

To investigate the effect of sST2 on BAT activation during cold exposure in BAT-denervated mice, we performed surgical denervation of BAT in both sST2-KO and WT littermates, after which these mice were subjected to cold exposure at 10 °C (Fig. 5c). Interestingly, compared with WT mice, BAT-denervated sST2-KO mice exhibited notable hypothermia development within 10 h of cold exposure. Consequently, their body temperature significantly decreased (Fig. 5e, f). Although the body weights of the sST2-KO mice remained unchanged, the weights of their BAT markedly increased (Fig. 5g, h). Notably, the BAT in sST2-KO mice tended to appear whiter (Fig. 5i). H&E staining demonstrated a substantial decrease in the characteristic multilocular structure of BAT, accompanied by the notable accumulation of lipid droplets (Fig. 5j). Electron microscopy (EM) revealed mitochondrial swelling and disrupted cristae within the BAT of sST2-KO mice, in contrast to the densely packed and intact cristae observed in control mice (Fig. 5k, l). More importantly, metabolic cage studies revealed that sST2-KO mice exhibited reduced oxygen consumption and energy expenditure, whereas food intake and locomotor activity remained unchanged (Fig. 5m; Supplementary Fig. S5b–d). No significant differences in lean mass or fat mass were detected between the two groups (Supplementary Fig. S5e). These results demonstrate that sST2 is required for maintaining BAT activation after BAT denervation.

RNA sequencing (RNA-seq) analysis revealed that in the BAT of sST2-KO mice after cold exposure, a total of 684 genes were upregulated, whereas 670 genes were downregulated (Fig. 5n). Subsequent gene set enrichment analysis (GSEA) revealed that the downregulated genes were predominantly linked to heat production (Fig. 5o, p). In contrast, the upregulated genes were largely involved in cytokine signaling pathways and various immune response mechanisms (Supplementary Fig. S5f). Importantly, qPCR analysis confirmed that the expression of thermogenic genes was significantly reduced in BAT from sST2-KO mice (Fig. 5q). Given that BAT thermogenesis relies on both UCP1-dependent and -independent mechanisms, we further examined the regulatory role of sST2 in the expression of UCP1-independent thermogenic genes. The results revealed that the expression of genes involved in UCP1-independent pathways, including creatine futile cycling, calcium futile cycling, the peroxisome pathway, and lipid futile cycling, remained unaltered (Supplementary Fig. S5g). Moreover, the phosphorylation levels of PKA substrates and HSL were significantly decreased in the BAT of sST2-KO mice (Fig. 5r). These findings strongly suggest that the KO of sST2 impairs the β-adrenergic-induced activation of UCP1-dependent BAT thermogenesis specifically in the context of depleted NE levels within BAT. Furthermore, sST2-KO mice exhibited a notable reduction in plasma NEFA levels, accompanied by a significant increase in TG levels, suggesting impairments in lipolysis and fatty acid utilization, which may be attributed to the diminished activity of the β-adrenergic signaling (Fig. 5s, t).

Next, we investigated the role of sST2 in BAT activation during cold exposure when NE was present in BAT. During the first 2 h of cold exposure at 4 °C, there was no significant difference in body temperature between sST2-KO mice and controls, likely because NE-driven thermogenesis dominates at this early stage. However, after 4 h of cold exposure, as the NE levels in the BAT decreased, the body temperature of the sST2-KO mice decreased more significantly than that of the control mice. After 10 h of cold exposure, a greater percentage of sST2-KO mice manifested clear signs of hypothermia (Supplementary Fig. S5h). Infrared imaging confirmed that sST2-KO mice had lower body temperatures after cold exposure (Supplementary Fig. S5i). Despite the fact that both the body and BAT weights remained unaltered following cold exposure (Supplementary Fig. S5j), the BAT in sST2-KO mice exhibited a pronounced whitish appearance (Supplementary Fig. S5k), and histological analysis revealed that there were larger lipid droplets in the BAT of these mice, which is consistent with the morphological changes observed in the mice after BAT denervation (Supplementary Fig. S5l). Metabolic cage analyses revealed that sST2 deficiency blunted the NE-stimulated increase in energy expenditure (Supplementary Fig. S5m), suggesting that NE-induced sST2 contributes to increased energy expenditure in mice. Additionally, in the BAT of sST2-KO mice, the mRNA and protein expression levels of thermogenic genes such as *Ucp1*, *Dio2*, and *Pgc1α* were markedly suppressed (Supplementary Fig. S5n, o), and the phosphorylation of PKA substrates and HSL was also reduced (Supplementary Fig. S5o). Lower NEFA levels and higher TG levels were detected in the plasma of sST2-KO mice after cold exposure, providing additional evidence for the importance of sST2 in regulating thermogenesis and metabolic homeostasis under physiological conditions (Supplementary Fig. S5p).

In summary, these results clearly demonstrate that sST2 is critically important for facilitating the activation of BAT via an endocrine mechanism that is essential for maintaining continuous thermogenesis during cold stress. Notably, this significance is particularly pronounced when the direct influence of NE on BAT is diminished.

### sST2 binds to ARs to activate BAT thermogenesis in an IL33-independent manner

**Fig. 6:**
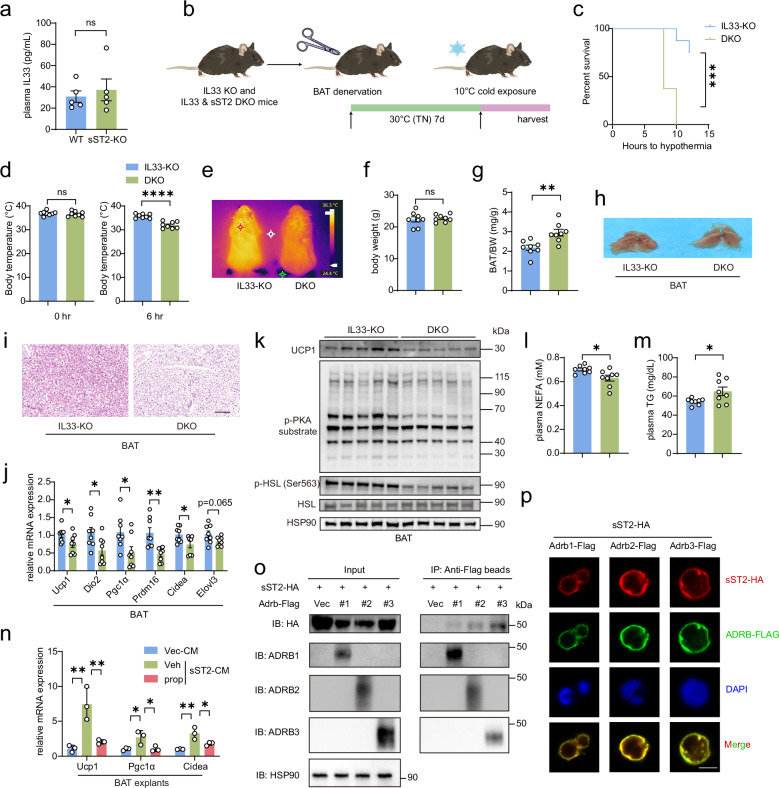
sST2 activates BAT thermogenesis by binding to adrenergic receptors, a process that is independent of IL-33. **a** Plasma IL-33 levels in 10-week-old sST2-KO mice and WT littermates (*n* = 5 per group). **b** Schematic illustrating 10-week-old IL-33 KO mice and sST2/IL-33 double-knockout (DKO) mice subjected to BAT denervation and exposed to cold stimulus at 10 °C for 12 h. **c**, **d** Survival curves (**c**) of IL-33 KO and DKO mice (*n* = 8 per group) subjected to BAT denervation and exposed to cold stimulus at 10 °C for 12 h. The core body temperature was recorded after 6 h of cold exposure (**d**). The mice were euthanized when their core temperature dropped below 30 °C. **e** Representative infrared thermography images of IL-33 KO and DKO mice after BAT denervation and exposure to cold stimulus at 10 °C for 8 h. **f**, **g** Body weights (**f**) and BAT weight-to-BW ratios (**g**) of IL-33 KO and DKO mice (*n* = 8 per group) after BAT denervation and exposure to cold stimulus at 10 °C for 8 h. **h**, **i** Gross morphology (**h**) and H&E staining (**i**) of BAT from IL-33 KO and DKO mice after BAT denervation and exposure to cold stimulus at 10 °C for 8 h. Scale bar, 100 μm. **j** qPCR analysis of the expression of thermogenic genes in BAT from IL-33 KO and DKO mice (*n* = 8 per group) after BAT denervation and exposure to cold stimulus at 10 °C for 8 h. **k** Immunoblotting analyses showing the levels of UCP1, phosphorylated PKA substrate, phosphorylated HSL and total HSL in BAT from IL-33 KO and DKO mice after BAT denervation and exposure to cold stimulus at 10 °C for 8 h. HSP90 was used as a loading control. **l**, **m** NEFA (**l**) and TG (**m**) levels in plasma from IL-33 KO and DKO mice (*n* = 8 per group) after BAT denervation and exposure to cold stimulus at 10 °C for 8 h. **n** qPCR analysis of the expression of thermogenic genes in BAT explants from WT mice treated with conditioned medium (CM) from HEK293T cells overexpressing vector (Vec) or sST2 combined with vehicle (Veh) or propranolol (prop, 1 µM) for 24 h (*n* = 3 per group). **o** Western blot analysis demonstrating co-immunoprecipitation of FLAG-tagged ADRB1, ADRB2, ADRB3 and HA-tagged sST2 in HEK293T cells following co-transfection with their respective plasmids. **p** Confocal microscopy images of FLAG (green), HA (red), and DAPI (blue) in HEK293T cells transfected with FLAG-tagged ADRB1, ADRB2, and ADRB3 and treated with CM from HEK293T cells expressing HA-tagged sST2 for 1 h. Scale bar, 10 μm. Data are shown as mean ± SEM. Statistical significance was determined by two-tailed unpaired Student’s *t*-test in **a**, **d**, **f**, **g**, **j**, **l**, **m**; one-way ANOVA with multiple comparisons tests in **n**; and the log-rank (Mantel–Cox) test in **c**. *P < 0.05, **P < 0.01, ****P < 0.0001; ns not significant.

sST2 functions as a decoy receptor of IL33, thereby inhibiting IL33 activity. Recent studies have demonstrated that increased IL-33 levels promote beige fat formation^51^. In our study, however, we observed that depletion of sST2 did not significantly affect plasma IL33 levels, suggesting that the role of sST2 in inducing BAT thermogenesis may be independent of IL33 activity (Fig. 6a).

To investigate this hypothesis, we used an IL33-KO mouse line in which plasma IL33 levels were significantly decreased (Supplementary Fig. S6a). We then crossed sST2-KO mice with IL33-KO mice to generate an sST2/IL33 double-knockout (DKO) mouse model and subjected the DKO mice to BAT denervation. Moreover, IL33 single-KO mice were utilized as controls (Fig. 6b). After the mice were subjected to cold exposure at 10 °C, the DKO mice exhibited a pronounced cold-sensitive phenotype. The percentage of hypothermic DKO mice was markedly greater than that of IL33 single-KO mice. In addition, DKO mice exhibited significantly lower body temperatures (Fig. 6c–e). Furthermore, the BAT of the DKO mice was heavier and whiter in color and exhibited increased lipid droplet accumulation (Fig. 6f–i). Notably, the expression of thermogenic genes in BAT was attenuated, and the phosphorylation of PKA substrates and HSL was reduced, indicating impaired activation of β-adrenergic signaling in the BAT of DKO mice (Fig. 6j, k). In addition, in DKO mice, plasma NEFA levels were lower, while TG levels were higher, suggesting that lipolysis and fatty acid utilization were inhibited (Fig. 6l, m). Overall, the phenotype of BAT-denervated DKO mice after cold exposure was similar to that of sST2 single-KO mice. These findings suggest that sST2 may trigger the activation of BAT via an IL33-independent mechanism.

We previously found that recombinant sST2 directly activates β-adrenergic signaling in BACs, possibly through the activation of β-AR (Fig. 2c, d). Furthermore, treatment with propranolol, a β-AR antagonist, counteracts the upregulation of thermogenic gene expression in BAT explants upon treatment with sST2 conditioned medium (Fig. 6n). On the basis of these findings, we hypothesize that sST2 may directly bind to β-AR. To validate this hypothesis, we conducted immunoprecipitation assays to precisely assess the interaction between sST2 and the three major β-AR isoforms ADRB1, ADRB2, and ADRB3. Our results revealed that sST2 interacts with all three ARs. Specifically, the interaction with ADRB3 was the most robust, while the interaction with ADRB1 was the weakest (Fig. 6o). Immunofluorescence assays conducted in 293 T cells provided conclusive evidence of the colocalization of overexpressed sST2 and β-AR on the cell membrane. This observation highlights the intriguing possibility that sST2 functions as a novel humoral ligand of β-AR (Fig. 6p).

Collectively, our findings strongly suggest that sST2 triggers BAT thermogenesis by directly binding to β-AR independent of IL33. To validate this hypothesis, we administered the selective ADRB3 agonist CL316,243 to DKO and IL33 single-KO mice after BAT denervation but before 10 °C cold exposure (Supplementary Fig. S6b). Notably, cold intolerance and impaired thermogenesis were significantly ameliorated in DKO mice. After treatment, no significant differences were detected between DKO mice and control mice in terms of thermogenic capacity (Supplementary Fig. S6c–e). Moreover, neither body weight nor BAT weight significantly changed following treatment (Supplementary Fig. S6f, g). Gross morphological inspection and H&E staining indicated that the BAT was fully activated in both groups (Supplementary Fig. S6h, i). Additionally, thermogenic gene expression and adrenergic signaling activity in the BAT of DKO mice were completely restored (Supplementary Fig. S6j, k). These findings offer compelling evidence that impaired activation of β-AR plays a predominant role in the inhibition of BAT thermogenesis observed in BAT-denervated sST2-KO mice.

### Overexpression of sST2 cell-autonomously drives beige fat formation in mice and humans in a IL33-independent manner

Activation of thermogenic fat is a promising strategy for treating obesity and associated metabolic disorders. The ADRB3 agonist mirabegron has been shown to improve insulin resistance and mitigate the progression of obesity in humans^38,39^. Since sST2 triggers BAT thermogenesis independent of IL33 by binding to β-AR, predominantly ADRB3, we investigated whether sST2 could synergize with ADRB3 agonists to boost thermogenic fat activation.

**Fig. 7:**
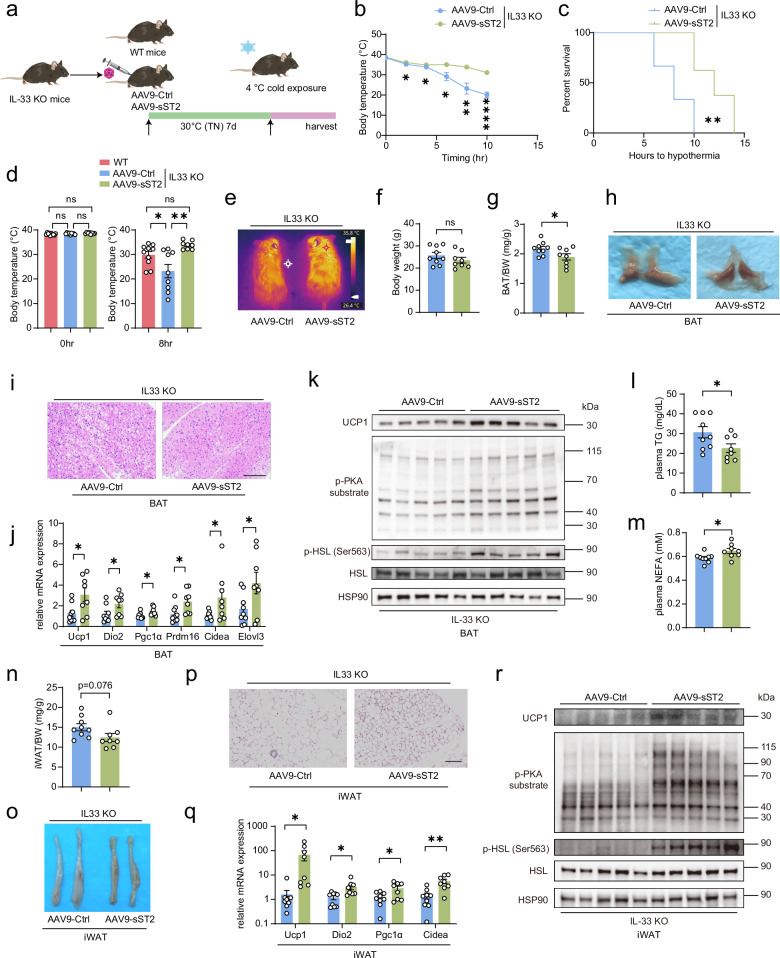
sST2 overexpression promotes beige fat formation upon cold exposure, which is independent of IL-33 signaling. **a** Schematic depicting 10-week-old IL-33-KO mice subjected to tail vein injection of AAV followed by exposure to cold stimulus at 4 °C for 14 h. **b**, **c** Line charts depicting changes in core body temperature at the indicated time points (**b**) and survival curves (**c**) of IL-33-KO mice after tail vein injection of AAV9-Ctrl or AAV9-sST2, followed by exposure to cold stimulus at 4 °C for 14 h (*n* = 8–9 per group). The mice were euthanized when their core temperature dropped below 30 °C. **d** Core body temperature of WT mice and IL33-KO mice after tail vein injection of AAV9-Ctrl and AAV9-sST2, followed by exposure to cold stimulus at 4 °C for 8 h (*n* = 8–10 per group). **e** Representative infrared thermography images of IL-33-KO mice intravenously injected with AAV9-Ctrl or AAV9-sST2 and exposed to cold stimulus at 4 °C for 8 h. **f**, **g** Body weights (**f**) and the BAT weight-to-BW ratios (**g**) of IL33-KO mice intravenously injected with AAV9-Ctrl and AAV9-sST2 and exposed to cold stimulus at 4 °C for 8 h (*n* = 8–9 per group). **h**, **i** Gross morphology (**h**) and H&E staining (**i**) of BAT from IL33-KO mice after AAV9-Ctrl and AAV9-sST2 injection and 4 °C cold exposure for 8 h. Scale bar, 100 μm. **j** qPCR analysis of the expression of thermogenic genes in BAT from IL33-KO mice after AAV9-Ctrl and AAV9-sST2 injection and 4 °C cold exposure for 8 h (*n* = 8–9 per group). **k** Immunoblotting showing the levels of UCP1, phosphorylated PKA substrate, phosphorylated HSL and total HSL in BAT from IL33-KO mice after AAV9-Ctrl and AAV9-sST2 injection and 4 °C cold exposure for 8 h. HSP90 was used as a loading control. **l**, **m** NEFA (**l**) and TG (**m**) levels in plasma from IL-33-KO mice after AAV9-Ctrl and AAV9-sST2 injection and 4 °C cold exposure for 8 h. **n**–**p** iWAT weight to BW ratio (**n**) gross morphology (**o**) and H&E staining (**p**) of IL33-KO mice intravenously injected with AAV9-Ctrl and AAV9-sST2 and exposed to cold stimulus at 4 °C for 8 h (*n* = 8–9 per group). Scale bar, 100 μm. **q** qPCR analysis of the expression of thermogenic genes in iWAT from IL33-KO mice after AAV9-Ctrl and AAV9-sST2 injection and 4 °C cold exposure for 8 h (*n* = 8–9 per group). **r** Immunoblotting showing the levels of UCP1, phosphorylated PKA substrate, phosphorylated HSL and total HSL in iWAT from IL33-KO mice after AAV9-Ctrl and AAV9-sST2 injection and 4 °C cold exposure for 8 h. HSP90 was used as a loading control. Data are shown as mean ± SEM. Statistical significance was determined by two-tailed unpaired Student’s *t*-test in **f**, **g**, **j**, **l**–**n**, **q**; two-way ANOVA multiple comparison tests in **b**; one-way ANOVA multiple comparison tests in **d**; and log-rank (Mantel–Cox) tests in **c**. *P < 0.05, **P < 0.01, ****P < 0.0001; ns not significant.

Previous studies have demonstrated that IL33 promotes beige fat formation. Given that sST2 both activates β-adrenergic signaling and inhibits IL33 activity, we overexpressed sST2 in both WT and IL33-deficient (IL33-KO) mice using adeno-associated viruses (AAVs) and subjected these mice to cold exposure. This approach allowed us to assess the role of sST2 in thermogenic fat formation in both the presence and absence of IL33 (Fig. 7a; Supplementary Fig. S7a).

We first confirmed that AAV-mediated overexpression of sST2 significantly increased plasma sST2 levels without affecting IL33 levels in WT mice (Supplementary Fig. S7b, c). In the IL33-KO background, the body temperature of mice overexpressing sST2 remained similar to that of control mice under room temperature conditions but was significantly higher upon cold exposure. Additionally, mice overexpressing sST2 were more resistant to cold stress (Fig. 7b, c). Compared with that of WT mice, the body temperature of IL33-KO mice markedly decreased (Fig. 7d). Notably, in sST2-overexpressing mice, the hypothermia caused by IL33 deficiency was completely reversed, and this effect was validated by infrared imaging of IL33-KO mice (Fig. 7d, e). In contrast, in mice overexpressing sST2 in the WT background, no significant difference in body temperature was observed (Supplementary Fig. S7d, e). Interestingly, overexpression of sST2 did not affect the body weight of either WT or IL33-KO mice but significantly decreased BAT weight in IL33-KO mice (Fig. 7f, g; Supplementary Fig. S7f, g). Additionally, the BAT of sST2-overexpressing IL33-KO mice exhibited a browner phenotype. Histological analysis confirmed an increase in the multilocular lipid content, signifying enhanced browning. However, the BAT of sST2-overexpressing WT mice did not display this phenotype (Fig. 7h, I; Supplementary Fig. S7h, i). Consistent with these findings, thermogenic gene expression and β-adrenergic signaling activation were strongly elevated in the BAT of sST2-overexpressing IL33-KO mice. In comparison, WT mice overexpressing sST2 presented relatively minor changes in thermogenic gene expression and β-adrenergic signaling (Fig. 7j, k; Supplementary Fig. S7j, k). Furthermore, plasma metabolite analysis revealed that the overexpression of sST2 in IL33-KO mice decreased TG levels but increased NEFA levels. Furthermore, the overexpression of sST2 in WT mice led to an increase in the NEFA levels only (Fig. 7l, m; Supplementary Fig. S7l, m). These data indicate that sST2 can promote the activation of the adrenergic pathway in BAT, regardless of IL33 expression. Notably, the effect of sST2 on BAT activation markedly differed between WT and IL33-KO mice. We speculate that in IL33-deficient mice, low basal BAT activity may provide a conducive environment for sST2 to mediate a more substantial effect. Conversely, in WT mice, the well-established NE-driven BAT activation pathway overpowers the potential effects of sST2. However, our experimental evidence revealed that in both WT and IL33-KO mice, overexpression of sST2 led to a significant increase in beige fat formation. This was clearly demonstrated by the general morphology and H&E staining of the inguinal white adipose tissue (iWAT), which revealed characteristic morphological changes indicative of beige fat development (Fig. 7n–p; Supplementary Fig. S7n–p). Additionally, the upregulation of thermogenic gene expression in iWAT further supported this conclusion, underscoring the ability of sST2 overexpression to potently induce the formation of beige fat (Fig. 7q, r; Supplementary Fig. S7q, r). Mechanistically, β-adrenergic activation in iWAT likely drove this effect (Fig. 7r; Supplementary Fig. S7r). Importantly, sST2 overexpression induced a comparable degree of iWAT beiging in both WT and IL33-KO mice. These findings suggest that the sST2-mediated inhibition of endogenous IL33 does not impede the direct role of sST2 in driving beige fat formation.

**Fig. 8:**
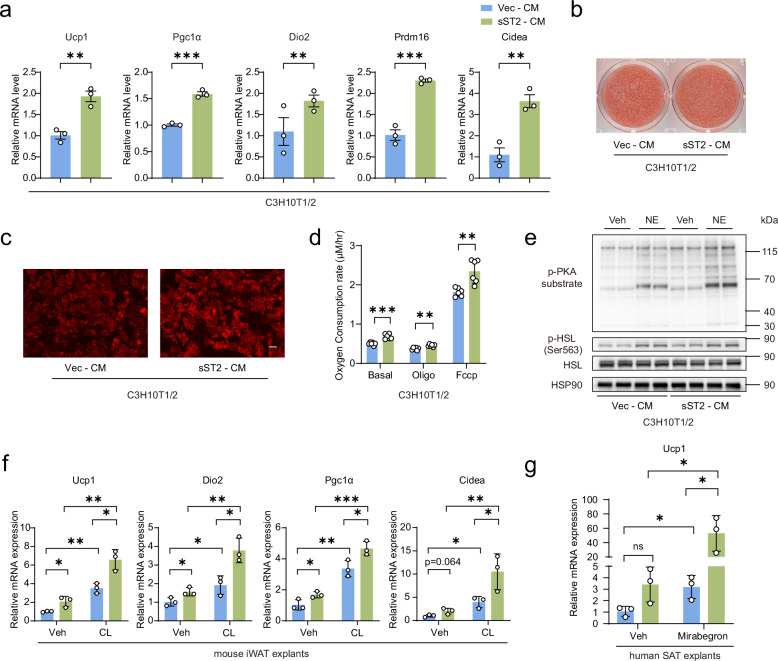
sST2 treatment induces beige adipogenesis in synergy with β-adrenergic stimuli. **a** qPCR analysis of the expression of thermogenic genes in C3H10T1/2 cells treated with vector (Vec) or sST2-conditioned medium (CM) during differentiation (*n* = 3 per group). **b**, **c** Oil red O (**b**) and MitoTracker staining (**c**) of C3H10T1/2 cells treated with Vec and sST2 CM during differentiation. Scale bar, 100 μm. **d** Oxygen consumption rate (OCR) of differentiated C3H10T1/2 cells treated with Vec and sST2 CM during differentiation. FCCP (10 µM) and oligomycin (Oligo, 10 µg/mL) were used to evaluate mitochondrial respiratory function (*n* = 6 per group). **e** Western blot analyses of phosphorylation of PKA substrate and HSL in differentiated C3H10T1/2 cells treated with Vec and sST2 CM during differentiation. NE (1 µM) was applied for 15 min before sample collection. HSP90 was used as a loading control. **f** qPCR analysis of the expression of thermogenic genes in iWAT explants from WT mice treated with Vec and sST2 CM in combination with vehicle (Veh) or CL 316,243 (CL, 1 µM) for 24 h (*n* = 3 per group). **g** qPCR analysis of UCP1 expression in human subcutaneous adipose tissue (SAT) explants treated with Vec and sST2 CM in combination with Veh or mirabegron (1 µM) for 24 h (*n* = 3 per group). Data are shown as mean ± SEM. Statistical significance was determined by two-tailed unpaired Student’s *t*-test in **a**, **d**; and one-way ANOVA for multiple comparisons in **f**, **g**. *P < 0.05, **P < 0.01, ***P < 0.001; ns not significant.

To explore whether sST2 treatment promotes beige or BAC differentiation, we treated C3H10T1/2 cells and BACs with sST2-containing conditioned medium (Supplementary Fig. S8a). Treatment with sST2 conditioned medium directly induced the expression of thermogenic genes in these two cell lines (Fig. 8a; Supplementary Fig. S8b). Although Oil Red O staining indicated that sST2 treatment did not cause any significant changes in lipid deposition in differentiated C3H10T1/2 cells or BACs, mitochondrial staining revealed a notable increase in mitochondrial mass within these cells (Fig. 8b, c; Supplementary Fig. S8c, d). In addition, compared with control cells, sST2-treated C3H10T1/2 cells and BACs exhibited a higher mitochondrial respiration rate (Fig. 8d; Supplementary Fig. S8e). Consistent with previous results, sST2 treatment led to a significant enhancement of β-adrenergic signaling in C3H10T1/2 cells (Fig. 8e). These results strongly suggest that sST2 promotes the differentiation of C3H10T1/2 cells toward a beige phenotype via the activation of β-adrenergic signaling.

Although mirabegron has been developed as a human ADRB3 agonist, the high doses needed restrict its clinical applicability. Previous findings suggested that sST2 specifically targets and acts upon ADRB3. To overcome this shortcoming, we investigated whether sST2 synergized with ADRB3 agonists to induce thermogenic gene expression. The ultimate goal was to employ mirabegron or alternative ADRB3 agonists at low dosages, thereby circumventing the adverse effects commonly associated with high-dose administration. In subcutaneous adipose tissue explants obtained from both murine and human subjects, treatment with CL316,243 and mirabegron led to the significant upregulation of thermogenic gene expression. Notably, this stimulatory effect synergized with sST2. These findings suggest that sST2 can serve as an enhancer to potentiate the effects of ADRB3 agonists in activating thermogenic fat (Fig. 8f, g). Given that sST2 is currently a well-recognized biomarker for heart failure, we next conducted a comprehensive assessment of the potential cardiovascular risks of sST2 treatment. In-depth analyses, including cardiac ultrasound imaging and quantification of heart failure-related markers, revealed that sST2 overexpression did not lead to any detectable impairment of normal cardiac function in murine models and that β-adrenergic signaling in the heart was not activated (Supplementary Fig. S8f–i). Based on these findings, we propose that the combined use of ADRB3 agonists and sST2 might present a novel and effective approach for promoting the activation of thermogenic fat in both murine and human subjects.

## Discussion

The activation of BAT thermogenesis during cold exposure serves as a crucial defense mechanism against cold environments. Catecholamines, particularly NE released from sympathetic nerves, play a central role in this process. In this study, we carefully monitored dynamic changes in NE levels within BAT in response to acute cold exposure. Unexpectedly, we observed a temporal discrepancy between NE levels and the extent of BAT activation, suggesting the presence of alternative cold-sensing mechanisms that may sustain BAT thermogenesis under prolonged cold conditions as a substitute for NE.

Previous studies have indicated that other neuromodulators, such as NPY, may also contribute to BAT activation^19^. To explore whether this alternative mechanism is neural or humoral, we conducted BAT denervation experiments. By blocking neurotransmitter release from the sympathetic nerves innervating BAT, we found that sympathetic nerves are essential for maintaining body temperature during the initial 2 h of cold exposure. However, under mild cold conditions (10 °C), BAT-denervated mice were still capable of activating BAT thermogenesis and β-adrenergic signaling in the absence of NE. These findings strongly suggest the involvement of a humoral mechanism in BAT activation that is independent of neurotransmitter stimulation.

To identify potential humoral mediators, we performed a plasma proteomic analysis and identified sST2, an adipokine derived from eWAT, as a key candidate. Several lines of evidence support this conclusion. First, both mass spectrometry and ELISA revealed significantly elevated sST2 levels in BAT-denervated mice. In WT mice, sST2 levels gradually increased following cold exposure and were correlated inversely with NE levels and directly with BAT activation. Second, sST2 expression in eWAT mirrored that in plasma. Notably, sST2 expression and secretion in adipocytes were directly induced by NE or ISO. Third, exogenous administration of sST2 activated β-adrenergic signaling in BAT, which is critical for thermogenic gene expression during cold exposure. Finally, cold exposure activated β1/2-adrenergic signaling, which upregulated Creb1 expression and subsequently promoted sST2 expression. While previous studies have demonstrated that cold-induced lipolysis activates oleic acid-Gpr3 signaling, which in turn induces PKA activation and thermogenic gene expression, our study suggests that this pathway plays a minor role in physiological BAT activation upon cold exposure. This is attributed to the significantly lower expression of the Gpr3 receptor than that of ARs in BAT.

Historically, animal models have targeted ST2 globally, leading to concurrent depletion of both the membrane-bound ST2L and the soluble isoform sST2. This confounding effect has impeded efforts to elucidate the specific role of sST2. In this study, we employed a novel genetic strategy to selectively eliminate sST2 while preserving normal ST2L expression. Using this refined model, we demonstrated that sST2 is essential for maintaining BAT thermogenesis under NE-deficient conditions and is required for prolonged thermogenic activation during extended cold exposure. Importantly, these functions are independent of IL-33. Although sST2 is widely regarded as a decoy receptor for IL-33, most previous research has focused on its immunomodulatory role in sequestering IL-33, and the biological functions of sST2 itself have remained largely unexplored. From an evolutionary standpoint, compared with IL-33, ST2 appeared earlier and is more conserved, implying that ST2 may play fundamental biological roles that extend beyond the role in the IL-33 axis^66^. Our study revealed that sST2 directly interacts with β-ARs, particularly ADRB3, to promote BAT activation and trigger β-adrenergic signaling. This previously unrecognized mechanism highlights the IL-33-independent roles of sST2 in thermogenic regulation and potentially other physiological and pathological contexts.

Taken together, our findings reveal a novel WAT-to-BAT regulatory axis mediated by sST2. This axis responds to neural stimuli, functions as a humoral pathway to drive β-adrenergic signaling activation in BAT, and thereby maintains thermogenic activity in BAT when NE levels decline. These results underscore the critical role of this WAT-derived adipokine in mediating inter-tissue communication with BAT to promote BAT activation during cold exposure. Moreover, this mechanism may synergize with neural regulation to dynamically modulate the progressive increase in BAT thermogenesis in response to cold stress.

Furthermore, we found that sST2 acts synergistically with ADRB3 agonists such as CL316,243 and mirabegron, enhancing the induction of beige adipocyte phenotypes in both murine and human adipose tissue explants. Despite its potential to sequester IL-33, sST2-induced beige fat formation was comparable between WT and IL-33-deficient mice. Importantly, sST2 administration exerted minimal effects on cardiac β-adrenergic signaling and did not impair cardiac function. Collectively, our results suggest that combining sST2 with ADRB3 agonists may represent a promising strategy to enhance thermogenic fat activity. This approach could increase efficacy while mitigating the cardiovascular side effects commonly associated with systemic β-adrenergic activation, thereby representing a safer and more effective option for obesity management.

## Limitations

A key limitation of this work is that our phenotypic and mechanistic analyses are restricted to physiological cold-stimulated thermogenesis, and extensive systemic metabolic characterization in pathological metabolic states is lacking. Notably, in metabolic disorders, elevated circulating levels of sST2 are significantly associated with the onset of diabetes and related metabolic features^67^. Our findings suggest that sST2 upregulation may constitute a compensatory response aimed at counteracting metabolic disease progression. Moreover, the functional effects of sST2 in this setting appear to be independent of the canonical IL-33/ST2L signaling pathway. Future studies will be needed to examine the role of this pathway in obese and diabetic animal models to define its impact on systemic energy balance and metabolic health and to explore its translational relevance for human metabolic disorders. These efforts will build upon the present findings and establish a more complete understanding of sST2 function in both physiological and pathophysiological contexts.

## Materials and methods

### Human samples

Plasma samples were obtained from patients admitted to the Cardiology Department of Shanghai Chest Hospital for treatment programs involving β-blockers. Initially, patients with no prior history of β-blocker use were selected for screening. Following confirmation from the attending physicians regarding their treatment plans, patients prescribed β-blockers were selected as the final cohort for inclusion. After informed consent was obtained, 3–5 mL of venous blood was collected at two time points: prior to the initiation of β-blocker treatment upon hospital admission and after 3–4 weeks of β-blocker treatment. Blood samples were collected into EDTA anticoagulant tubes and centrifuged at 4000 rpm for 5 min at 4 °C, after which the plasma supernatant was aspirated. The plasma was aliquoted into appropriate volumes and stored at –80 °C for subsequent analysis. Visceral adipose tissue (VAT) and subcutaneous adipose tissue (SAT) samples were obtained from patients who underwent surgery at the Cardiology Department of Shanghai Chest Hospital. These tissues were collected from excess VAT and SAT discarded during surgery and preserved in normal saline at 4 °C for use in subsequent experiments.

### Cell lines and conditioned medium preparation

All the cell lines were confirmed to be mycoplasma free. HEK293T, C3H10T1/2, and primary brown preadipocytes were cultured in complete medium containing high-glucose Dulbecco’s modified Eagle’s medium (DMEM) (BasalMedia, L110KJ), 10% fetal bovine serum (FBS) (Sigma, F8318) and 1% penicillin‒streptomycin (BasalMedia, S110JV) at 37 °C with 5% CO_2_. The medium was changed every 48 h until the cells reached confluence, at which point they were passaged at a 1:3 ratio. To prepare the sST2-containing conditioned medium, HEK293T cells were transfected with the pcDNA3-sST2 plasmids using polyethylenimine (PEI) (Shanghai Maokang, MX2202) and UltraFectin (BasalMedia, L530KJ), and cells transfected with the empty vector served as controls. After 12 h of incubation, the culture medium was replaced with fresh complete medium. The conditioned medium was collected 48 h post transfection and subsequently used to treat adipocytes and adipose tissue explants. All the cell culture experiments were independently repeated three times.

### Animals

All the mice used in this study had a C57BL/6J genetic background. WT C57BL/6J mice were purchased from Shanghai Lingchang Biotechnology. IL33-KO mice (NM-KO-190436) were obtained from Shanghai Model Organisms. sST2-KO mice were generated using a knock-in strategy resulting in deletion of introns 8–9 of the *Il1rl1* gene via the CRISPR/Cas9 system by GemPharmatech. The KO status was confirmed by PCR. KO mice were then crossed with WT mice to obtain heterozygous mice, and heterozygous mice were subsequently crossed to generate homozygous mice and WT mice, which were used as controls to ensure that there was no genetic background bias. IL33 and sST2 DKO mice were generated in a similar manner. All animal procedures were conducted in accordance with the protocols approved by the Institutional Animal Care and Use Committee (IACUC) of Shanghai Jiao Tong University School of Medicine (SJTU-SM), and this study was approved by the IACUC of SJTU-SM (#A2022-064). The mice were housed on a 12-h light/12-h dark cycle and provided standard rodent chow. Following guidelines for rodent euthanasia, plasma and tissues were harvested for analysis. Plasma NEFAs were detected using the LabAssay NEFA Detection Kit (Wako, 294-63601), and plasma TG levels were measured using the Serum Triglyceride Determination Kit (Sigma, TR0100) according to the manufacturer’s instructions. All the mouse experiments were independently repeated at least twice, with no animals excluded unless technical issues or human errors occurred. Our study examined 10-week-old male mice because male animals exhibit less phenotypic variability.

### Cold exposure mouse model

After a one-week acclimatization period, each 10-week-old C57BL/6 mouse was randomly assigned to one of two groups. The mice in the experimental group were placed into precooled cages, provided with food and water, and kept in a thermostatic chamber (Shanghai Bluepard, MGC-450HP-2) set to either 4 °C or 10 °C with 50% humidity for the designated duration. The mice in the control group were kept at room temperature. Core body temperature, measured rectally, was monitored at pre-determined time intervals throughout the cold exposure period using a rectal thermometer (Shanghai alcbio, 9238676). Infrared thermal images were captured using an infrared thermal camera (IRay Technology, M600F) to assess surface temperature. The mice were euthanized if their rectal temperature dropped below 30 °C, which was the established cutoff point for calculating the homeothermic fraction and generating survival curves. Both an infrared thermal camera and a rectal thermometer were used to measure the surface and core temperatures of the mice.

### Surgical denervation of BAT and removal of eWAT in mice

After acclimatization for one week, 10-week-old C57BL/6 mice were randomly assigned to two groups. One group underwent a sham surgery as a control, while the other group was subjected to sympathetic denervation of the BAT. The mice were anesthetized with 3% isoflurane for induction and maintained with 1.5% isoflurane (RWD, R510-22-10) mixed with oxygen supplied at 0.5 L/min. The surgical site was shaved, and the area was disinfected with sterile gauze soaked in iodine tincture. A transverse incision of ~0.5–1 cm in length was made along the lower edge of the scapula to expose the two intrascapular BAT lobes. With careful manipulation, the inner and lower portions of each lobe were accessed to visualize the five intercostal nerves on each side that provide sympathetic and sensory innervation. These nerves were then carefully isolated using a microscope. The identified nerves were severed at two or more points, and the segments between the cuts were excised to ensure complete BAT denervation and minimize the potential for nerve regeneration. In the sham surgery group, the fat pads were exposed, and the nerves were identified but left intact without any dissection. For eWAT removal, a vertical incision of ~0.5–1 cm in length was made along the medioventral line. Blood vessels were carefully ligated to prevent bleeding, and both sides of the eWAT were removed while ensuring that the epididymis was not damaged. In the sham surgery group, bilateral eWAT was gently pulled out and then replaced through the same abdominal incision. Following the surgical procedures for BAT denervation and eWAT removal, the skin incision was closed using aseptic wound clips, and the surgical site was disinfected once more with sterile gauze soaked in iodine tincture.

### Drug-induced adrenergic activation mouse models

Following acclimatization for one week, 10-week-old C57BL/6 mice were randomly assigned to two groups. The control group received intraperitoneal (i.p.) injections of sterile normal saline, while the experimental group was administered ISO (dissolved in sterile normal saline) (Sigma‒Aldrich, I5627) to induce adrenergic activation. Mice were weighed prior to administration, and ISO was administered at 30 mg/kg/day for 2 consecutive weeks or as a single acute injection of 100 mg/kg to simulate enhanced adrenergic stimulation and cold stress. To assess the reversibility of adrenergic activation, propranolol (MCE, HY-B0573), a non-selective β-AR antagonist, was administered at a dosage of 30 mg/kg/day. For specific stimulation of β3-adrenergic signaling, CL316,243 (Sigma‒Aldrich, C5976), a selective ADRB3 agonist, was administered at a dose of 30 mg/kg to bypass ADRB1 and ADRB2 activation. Throughout the treatment period, all the mice were housed at room temperature under a 12-h light/dark cycle with ad libitum access to standard chow and water. At the end of the treatment regimen, the animals were euthanized, and the relevant tissues were collected for further analysis.

### sST2-overexpressing mouse models

An AAV carrying sST2 was constructed under the control of the CAG promoter. AAV-CAG-MCS plasmids were used as destination vectors for AAV construction. The sST2 sequence was amplified from cDNA obtained from C57BL/6 WT mouse tissue and inserted into the AAV-CAG-MCS plasmid. HEK293T cells were cultured in 30 cm × 15 cm plates until they reached 80% confluence and then transfected with either AAV-CAG-GFP or AAV-CAG-sST2 plasmids, along with AAV helper plasmid and packaging plasmids (pAdΔF6 and pAAV9), using PEI in high-glucose DMEM. At 60 h post transfection, viral particles were harvested and lysed with 2.5 mL of lysis buffer (150 mM NaCl and 20 mM Tris-HCl, pH 8.0). The lysate was subjected to three freeze-thaw cycles to ensure complete lysis. To degrade residual DNA, 5 mL of 1 M MgCl_2_ and 5 μL of 250 KU/mL SuperNuclease (Sino Biological, SSNP01) were added, and the mixture was incubated at 37 °C for 15 min. The viral particles were purified using discontinuous iodixanol gradient centrifugation (17%-25%-40%-60% OptiDensity) (BasalMedia, R714JV), 1 mM MgCl_2_, 2.5 mM KCl, and 1 M NaCl) in an OptiSeal tube (Beckman, 342413) at 53,000 rpm and 14 °C for 3 h. Approximately 1 mL of the 40% iodixanol layer, containing the viral fraction, was collected using a syringe. The virus was washed and concentrated with PBS (BasalMedia, B320KJ) using an Amicon Ultra Filter (Millipore, UFC910096), and the final volume was reduced to ~500 μL. Viral titers were determined by qPCR. Mice were injected with AAV9-sST2 (2 × 10^11^ viral particles per mouse) via the caudal vein and housed at a neutral temperature (30 °C) prior to cold exposure to ensure stability. AAV9-GFP was used in the comparison group.

### Body composition analysis and metabolic cage studies

Body composition, including fat mass and lean mass, was measured in chow-fed mice using an EchoMRI-100H analyzer (Echo Medical Systems). Indirect calorimetry was performed with a Comprehensive Laboratory Animal Monitoring System (CLAMS; Columbus Instruments) at ambient room temperature. The mice were individually housed in metabolic chambers and acclimated for 24 h prior to data collection, followed by a 48-h monitoring period under a 12-h light/dark cycle with ad libitum access to a chow diet and water. The oxygen consumption, carbon dioxide production and heat production were normalized to the body weight of each mouse. Locomotor activity and food intake were simultaneously monitored using the integrated sensors in each chamber. Regression-based analyses of the relationship between energy expenditure and body weight were performed using the CalR web-based analysis tool (https://calrapp.org). To assess the energy expenditure induced by NE, the mice were anesthetized by i.p. injection of pentobarbital sodium (90 mg/kg) to achieve a stable physiological state. The mice subsequently received an i.p. injection of NE (1 mg/kg), and their physiological parameters were continuously recorded for 2 h, as previously described^68,69^.

### Primary brown preadipocyte isolation and culture

BAT from the interscapular region of neonatal C57BL/6J mice was excised and finely minced using curved scissors in an ice-cold PBS-filled dish. Afterward, the minced tissue was transferred to a 50 mL tube containing isolation buffer (123 mM NaCl, 5 mM KCl, 1.3 mM CaCl_2_, 5 mM glucose (Sigma‒Aldrich, G5767), 100 mM HEPES, 1% penicillin‒streptomycin, and 4% FBS) supplemented with 1.5 mg/mL collagenase Type I (Worthington, LS004196) (1 mL per sample from six mice). The suspension was vortexed briefly to mix and incubated in a 37 °C water bath with gentle rotation at 90 rpm. The mixture was vortexed every 7–8 min for a total of 5 times to facilitate digestion. Following enzymatic digestion, 4 mL of cold complete medium was added to terminate the reaction. The resulting cell suspension was filtered through a 100 μm cell strainer and centrifuged at 1000 rpm for 5 min. The supernatant was discarded, and the cell pellet, consisting of primary brown preadipocytes, was resuspended in 1 mL of primary culture medium (high-glucose DMEM, 20% FBS, 1% penicillin‒streptomycin, and 20 mM HEPES (Sigma, H4034)). The cells were then seeded into 12-well plates and incubated overnight in an environment with 5% CO_2_. After incubation, the cells were washed twice with 3 mL of PBS, and 3 mL of fresh primary culture medium was added to maintain the culture. The medium was changed every 48 h until the cells reached contact inhibition. Once confluent, the cells were passaged at a 1:3 ratio.

### Adipogenesis induction and adipocyte differentiation

C3H10T1/2 cells and primary brown preadipocytes used for adipogenesis were seeded into appropriately sized culture plates. Once the cells reached confluence, differentiation was induced by supplementation of the complete medium with 0.5 mM 3-isobutyl-1-methylxanthine (IBMX) (ACROS, 228420050), 125 µM indomethacin (Sigma, I-7378), 1 µM dexamethasone (Sigma, D2915), 20 nM insulin (Sigma, I5500), and 1 nM triiodothyronine (T3) (Sigma, T2877). After 48 h, the induction medium was replaced with maintenance medium containing 20 nM insulin and 1 nM T3. The medium was subsequently replaced every 48 h until the cells fully differentiated into mature adipocytes, as evidenced by the accumulation of lipid droplets, which was typically observed 5–7 days post induction. For thermogenic activation, adipocytes were treated with 1 µM NE (Sigma, A9512) for 6 h before collection. All the cultures were maintained at 37 °C in a humidified incubator with 5% CO_2_.

### Adipose tissue explant isolation and culture

Human SAT, as well as murine eWAT, iWAT, and BAT, was harvested and preserved in normal saline at 4 °C. Immediately after collection, the samples were transferred to high-glucose DMEM supplemented with 1% penicillin‒streptomycin. Afterward, they were maintained at 4 °C. The adipose tissue was finely minced into fragments of ~2–3 mm in size. Next, the tissue suspension was passed through a 100 µm mesh filter, and the filtrate was discarded. The retained tissue fragments were rinsed twice with 45 mL of PBS to remove debris. After that, to initiate explant culture, a 6-well plate was prepared with M199 medium (BasalMedia, L640KJ) supplemented with 1% penicillin‒streptomycin, 1 nM dexamethasone, and 1 nM insulin. The washed adipose tissue fragments were evenly distributed into the wells, and the plate was gently agitated to ensure uniform dispersion. Explants were incubated overnight at 37 °C in an 8% CO_2_ atmosphere for stabilization. On the following day, the culture medium was replaced, and the explants were subjected to drug treatment as indicated before collection for analysis.

### Adipose tissue fractionation

Human VAT was harvested and preserved in PBS at 4 °C. VAT pads were transferred to 500 µL of HBSS (BasalMedia, B430KJ) and finely minced on ice using curved scissors. The minced tissue was then placed into a 50 mL conical tube containing 2.5 mL of digestion buffer (HBSS supplemented with 1% BSA (Yeasen, 36104ES25), 6.25 mM CaCl_2_, 5 mM glucose, and 1 µM adenosine (Acros, 164040050)). An additional 2.5 mL of collagenase solution (high-glucose DMEM supplemented with 1% BSA, 2.4 U/mL dispase II (Roche, 4942078001), 3 mg/mL collagenase Type I, and 10 mM CaCl_2_) was added to the mixture. Digestion was carried out in a 37 °C water bath with shaking for 30 min. Following enzymatic digestion, the suspension was passed through a 70 µm filter, and digestion was halted by adding cold complete medium. The sample was then centrifuged at 1000 rpm for 5 min. Mature adipocytes were separated as the floating fraction, and the SVF was collected as the pellet.

### Luciferase assay

Luciferase activity was assessed using pGL3-basic plasmids. The promoter region spanning –4949 bp to +974 bp of the *Il1rl1* gene, encoding sST2, was cloned into the pGL3-basic plasmid (pGL3-sST2-promoter-WT). To verify the functional relevance of specific motifs, targeted deletions (–3445 bp to –3437 bp and –3422 bp to –3414 bp) were introduced within the promoter region (pGL3-sST2-promoter-Mutant1 and pGL3-sST2-promoter-Mutant2) using a ClonExpress Kit (C116-01, Vazyme). The *Creb1* sequence was amplified from cDNA derived from C57BL/6 WT mouse tissues and inserted into the pcDNA3 plasmid (pcDNA3-Creb1). HEK293T cells were cultured to 50%–60% confluence in a 24-well plate. Once the cells reached the appropriate confluence, they were co-transfected with 50 ng of pGL3 plasmid and 200 ng of pcDNA3 plasmid per well using PEI. After 24 h of transfection, the culture medium was discarded, and the cells were washed with PBS. Luciferase activity was measured using a Luciferase Assay System (Promega, E1501) following the manufacturer’s instructions.

### Establishment of stable overexpression and knockdown cell lines

Retroviral vectors were constructed using pMSCV-puro plasmids as the transfer vector for overexpression. The coding sequences of *Ngp*, *Cgref1*, *Orm2*, *Creb1*, *Adrb1*, *Adrb2*, and *Adrb3* were amplified from cDNA derived from C57BL/6J WT mouse tissues and cloned into the pMSCV-puro plasmid. An empty vector served as the control. For subsequent retrovirus production, HEK293T cells were seeded in 10 cm plates and cultured to 80% confluence before transfection. Cells were co-transfected with the transfer plasmid expressing the target gene, an envelope plasmid (pVSV-G), and a packaging plasmid (pGag-Pol) using PEI in high-glucose DMEM. The culture medium was replaced with fresh complete medium 12–16 h post transfection, and the virus-containing supernatants were collected after 24 h. The target cell lines were incubated with a 1:1 mixture of virus-containing medium and complete medium supplemented with 10 µg/mL polybrene (Sigma‒Aldrich, H9268) to enhance infection efficiency. Cells were exposed to the viral medium overnight at 37 °C, after which the medium was replaced with fresh complete medium. Puromycin (InvivoGen, ant-pr-1) selection was initiated 48 h post infection, with the concentration adjusted appropriately for each cell line. The culture medium was replaced regularly until all the cells in the blank control group were eliminated, ensuring the successful establishment of stable overexpression cell lines. The efficiency of gene overexpression was confirmed by qPCR.

Retroviral vectors were constructed using pSuper-puro plasmids as the transfer vector for knockdown. shRNA sequences targeting *Creb1* were either designed using InvivoGen’s siRNA Wizard (https://www.invivogen.com/sirnawizard/) or selected from pre-designed shRNA libraries (Merck MISSION^®^ shRNA). The shRNA sequences are listed in Supplementary Table S1. A scrambled sequence (TTCTCCGAACGTGTCACGT) was used as a negative control. Afterward, the procedures for transfection, viral production, target cell infection, and puromycin selection were performed similarly to those used to establish stable overexpression cell lines. The efficiency of gene knockdown was confirmed by qPCR.

### Immunofluorescence

HEK293T cells seeded on glass slides were transfected with either pcDNA3-ADRB1/2/3-FLAG or pcDNA3-sST2-HA plasmids. After 48 h, the ADRB1/2/3-FLAG-expressing cells were treated with conditioned medium from sST2-HA-expressing cells. One hour later, the cells were fixed with 3.7% formaldehyde (Sangon, A501912) for 10 min, followed by three washes with PBS, each for 5 min. The cells were permeabilized and blocked with 0.2% Triton X-100 (Sangon, A600198-0500) and 3% BSA (Sangon, A500023) in PBS for 1 h. The slides were incubated overnight at 4 °C with rabbit anti-HA (Proteintech, 51064-2-AP, 1:100) and mouse anti-FLAG (Proteintech, 66008-4-Ig, 1:1000) antibodies. After incubation, the slides were washed three times with PBS containing 0.1% Tween 20 (Servicebio, GC204002) for 5 min each. Next, the slides were incubated with CoraLite488 anti-mouse (Proteintech, SA00013-1, 1:500) and CoraLite594 anti-rabbit (Proteintech, SA00013-4, 1:500) secondary antibodies at room temperature for 2 h in the dark. The slides were then washed three times with PBS for 5 min each. To stain the nuclei, the slides were incubated with 1 μg/mL 2-(4-amidinophenyl)-6-indolecarbamidine dihydrochloride (DAPI; Beyotime, C1002) in the dark at room temperature for 5 min and then washed three times with PBS for 5 min each. Finally, the slides were mounted with antifade mounting medium, and images were captured using a confocal microscope.

### H&E staining of mouse tissues

After the mice were euthanized, BAT, iWAT, and eWAT were harvested as previously described. Afterward, the tissues were fixed in 4% formaldehyde and processed by a series of dehydration steps using gradient ethanol (Shanghai Hushi, 10009218). The tissues were subsequently embedded in paraffin and cut into 4 µm sections. Afterward, the slides were dewaxed using dimethylbenzene (Shanghai Hushi, 10023418) and rehydrated through a series of decreasing ethanol concentrations. H&E staining was performed following the instructions of the Hematoxylin‒Eosin (H&E) HD Constant Dye Kit (Servicebio, G1076). After staining, the slides were dehydrated using absolute ethanol, normal butanol (Shanghai Hushi, 10005208), and dimethylbenzene and then mounted with Rhamsan gum (Servicebio, WG10004160). The stained tissues were examined under a microscope.

### Oil red O and MitoTracker staining

For Oil red O staining, C3H10T1/2 cells and BACs were fixed with 4% paraformaldehyde for 30 min at room temperature. Oil Red O (ORO) dye (Sigma, O0625) working solution was prepared by mixing the stock with double-distilled water at a 3:2 ratio, followed by filtration through a 0.45 µm filter. The cells were then washed three times with double-distilled water and stained with ORO dye for 2 h at room temperature. After staining, the cells were washed three times with double-distilled water. Images were captured using a scanner. For MitoTracker staining, MitoTracker (1 mM; Invitrogen, M7510) was diluted to 250 nM in high-glucose DMEM. C3H10T1/2 cells and BACs were washed three times with PBS and then incubated with MitoTracker dye for 30 min at 37 °C. Following incubation, the cells were washed three times with PBS and observed under a fluorescence microscope.

### Transmission electron microscopy

The mice were euthanized, and the BAT was dissected into 1–2 mm pieces. The tissue was fixed overnight at 4 °C in a mixture of 2.5% glutaraldehyde (Shanghai Hushi, 30092436) and 2% paraformaldehyde, followed by fixation in 1% osmium tetroxide (Sigma‒Aldrich, 201030). The samples were dehydrated through a graded series of ethanol (30%, 50%, 70%, 80%, 95%, and 100%) for 10 min at each step. The specimens were then infiltrated with Embed 812 resin (Electron Microscopy Sciences, 14900) for 6 h and embedded in capsules containing Embed 812, followed by polymerization at 60 °C for 48 h. The samples were sectioned to a thickness of 70–90 nm and stained with uranyl acetate (Sigma–Aldrich, 73943) and lead citrate (Sigma–Aldrich, 15326) for contrast. All the samples were finally observed using a transmission electron microscope (HITACHI, H-7650).

### RNA extraction and real-time PCR

Total RNA was extracted from cells and mouse tissues using TRI Reagent (Molecular Research Center, TR118) following the manufacturer’s protocol. One microgram of RNA was reverse transcribed using HiScript II Q RT SuperMix (Vazyme, R222-01), and qPCR was performed using SYBR Green (Bimake, B21202) with 0.25 µM primers in a final volume of 10 µL. Real-time PCR was conducted on a LightCycler 480 II (Roche). The relative mRNA expression levels of specific genes were quantified using the comparative ∆∆CT method and normalized to the expression level of the internal control RPLP0. The sequences of the primers used for specific gene expression analyses are listed in Supplementary Table S2.

### RNA-seq and data analysis

RNA was extracted as described above. RNA-seq was performed on RNA isolated from the BAT of WT mice and sST2-KO mice subjected to BAT denervation and cold exposure. RNA-seq was performed by Novogene. In brief, RNA quality was assessed using an RNA Nano 6000 Assay Kit on a Bioanalyzer 2100 system (Agilent Technologies). Total RNA was used for library preparation, wherein mRNA was purified using poly-T oligo-attached magnetic beads and fragmented for cDNA synthesis. First-strand cDNA synthesis was carried out using random hexamer primers and M-MuLV Reverse Transcriptase. Second-strand cDNA synthesis was then performed using DNA polymerase I and RNase H, followed by conversion of remaining overhangs to blunt ends via exonuclease/polymerase activities. After adenylation of the 3′ ends of the DNA fragments, adaptors with hairpin loop structures were ligated for hybridization. cDNA fragments between 370 bp and 420 bp in length were selected and purified using the AMPure XP system (Beckman Coulter). PCR amplification was performed using Phusion High-Fidelity DNA Polymerase, universal PCR primers, and an index primer. The PCR products were subsequently purified (AMPure XP system) and library quality was assessed on an Agilent Bioanalyzer 2100 system. The clustered, index-coded samples were sequenced on an Illumina NovaSeq platform with 150 bp paired-end reads. Data processing and visualization of the differentially expressed genes were carried out using R Studio (https://www.r-project.org/). Quality control was performed using FastQC. HISAT2 was used for alignment against the mouse reference genome (mm39), and htseq-count was used to count aligned reads in a BAM file. Differential expression analysis was performed using DESeq2. Genes with significant differences in expression by > 2-fold between the sST2-KO group and the WT group were selected for further analysis. Gene enrichment analysis was performed using Metascape (https://metascape.org/gp/index.html). GSEA (https://www.gsea-msigdb.org/gsea/index.jsp) was carried out using GSEA software, with genes ranked on the basis of their *P* values and fold changes.

### Mass spectrometry and data analysis

Blood samples were collected into EDTA anticoagulant tubes, followed by centrifugation at 4000 rpm for 5 min at 4 °C. The plasma supernatant was then carefully aspirated. To reduce the effects of high-abundance proteins in the plasma, pre-treatment was performed using High Select Top14 Abundant Protein Depletion Mini Spin (A36370; Thermo Scientific) and EasyPept DeeP (OSFP0002; Omicsolution) kits. For LC-MS/MS analysis, 10 μL of plasma sample was injected onto a reversed-phase microcapillary column (0.1 mm × 150 mm, packed with 5 μm 100 Å Magic C18 resin; Michrom Bioresources) using an autosampler (HTS-PAL, CTC Analytics) for analysis by an HPLC system (Paragram MS4, Michrom Bioresources) at a flow rate of 1 μL/min over 15 min. Peptides were separated by gradient elution at a flow rate of 0.5 μL/min using buffer A (2% acetonitrile (ACN) with 0.1% formic acid) and buffer B (98% ACN with 0.1% formic acid) over 90 min as follows: 0%–35% buffer B for 90 min, 80% buffer B for 8 min, 95% buffer B for 12 min, and 0% buffer B for 20 min. Mass spectrometry data were acquired using an LTQ XL™ Linear Ion Trap Mass Spectrometer (Thermo Fisher Scientific) coupled with an ADVANCE Captive Spray Source (Michrom Bioresources). The parameters for high-resolution MS survey scans (R = 100,000 at *m/z* 400) were in a range of *m/z* 350–1800 with 10^6^ ion accumulation through automatic gain control. Siloxane (*m/z* 445.120025) was used as an internal standard to calibrate mass accuracy. The detected proteins were compared and annotated with known secreted protein databases^70^, and the secreted proteins were selected for further analysis.

### Immunoblotting

For cell samples, lysates were prepared using RIPA buffer (50 mM Tris-HCl, pH 7.6, 150 mM NaCl, 25 mM NaF, 2 mM sodium orthovanadate (Macklin, T850322), 0.1% SDS (BBI, A500228), 1% NP40 (Sangon, A600385), 1% sodium deoxycholate (BBI, A600150), and 10% glycerol (Sangon, A600232-0500)) supplemented with a mixture of protease and phosphatase inhibitors (Bimake, B14002). The lysates were centrifuged at 12,000 rpm for 15 min to remove any precipitate. The supernatant was collected, and the protein concentration was determined using the Detergent Compatible Bradford Protein Quantification Kit (Vazyme, E211-01). For frozen mouse tissue samples, lysates were extracted using lysis buffer (50 mM Tris-HCl, pH 7.6, 130 mM NaCl, 5 mM NaF, 25 mM β-glycerophosphate (Sangon, A500486), 1 mM sodium orthovanadate, 10% glycerol, 1% Triton X-100, 1 mM DTT (BBI, A620058), and 1 mM PMSF (Beyotime, ST506)) supplemented with a mixture of protease and phosphatase inhibitors using a homogenizer (Shanghai Jingxin, JXFSTPRP-CL). The samples were incubated on ice for 30 min to ensure complete lysis and then centrifuged at 12,000 rpm for 15 min. The supernatant was collected, and the protein concentration was quantified using the Detergent Compatible Bradford Protein Quantification Kit. For immunoblotting, the tissue and cell lysates were denatured by the addition of 3× SDS-PAGE loading buffer (150 mM Tris-HCl, pH 6.8, 5% SDS, 20% glycerol, 200 mM DTT, and 10% β-mercaptoethanol (Shanghai Hushi, 80076928)), which was diluted to 1× to standardize concentrations, and then the samples were boiled at 98 °C for 15 min. Then, 30 µg of each sample was loaded onto SDS-PAGE gels. Protein separation was performed on Bis-Tris protein gels (Biotend, Gel442015), after which the proteins were transferred onto a 0.45 μm PVDF membrane (Cytiva, 10600023). Nonspecific binding sites were blocked by incubating the membrane with 3% non-fat milk (Beyotime, P0216) or BSA at room temperature for 1 h. The membrane was then incubated with the appropriate primary antibodies overnight at 4 °C and washed with TBS (Sangon, B548105) containing 0.05% Tween-20 (Servicebio, GC204002). The membrane was incubated with HRP-conjugated secondary antibodies (Proteintech, SA00001-1/2) at room temperature for 1 h and then washed again with TBS containing 0.05% Tween-20. Protein bands were visualized using High-sig ECL (Meilunbio, MA0187), and images were captured using Chemiluminescent Imaging System (Tanon, 4200). The antibodies used are listed in Supplementary Table S3.

### Co-immunoprecipitation

To verify the interaction between sST2 and ADRB1/2/3, HEK293T cells were seeded in 6-well plates and transfected upon reaching 80% confluency. For each well, 2 μg of the pcDNA3-sST2-HA plasmid was co-transfected with 2 μg of the pcDNA3-ADRB1-FLAG, pcDNA3-ADRB2-FLAG, or pcDNA3-ADRB3-FLAG plasmid using PEI and high-glucose DMEM. The control group was transfected with the empty pcDNA3 vector. After 48 h of transfection, the cells were lysed in 300 μL of RIPA buffer (50 mM Tris-HCl, pH 7.6, 150 mM NaCl, 25 mM NaF, 2 mM sodium orthovanadate, 1% NP40, 1% sodium deoxycholate, and 10% glycerol) supplemented with 1% protease and phosphatase inhibitor cocktail, and incubated on ice for 15 min. The lysates were subsequently sonicated and centrifuged at 12,000 rpm for 3 min at 4 °C to remove cellular debris. A 50 μL aliquot of the lysate from each sample was mixed with 25 μL of 3× SDS-PAGE loading buffer (150 mM Tris-HCl, pH 6.8, 5% SDS, 20% glycerol, 200 mM DTT, and 10% β-mercaptoethanol) to serve as the input sample. The remaining lysate was incubated with 10 μL of anti-DYKDDDDK magnetic agarose (Thermo Scientific, A36797) at 4 °C with rotation at 16 rpm for 4–6 h. The samples were subsequently washed five times with wash buffer (50 mM Tris-HCl, pH 8.0, 150 mM NaCl, 1 mM MgCl_2_, and 0.5% NP40), incubated with 80 μL of 1× SDS-PAGE sample buffer (50 mM Tris-HCl, pH 6.8, 1.5% SDS, 10% glycerol, 100 mM DTT, and 5% β-mercaptoethanol), and heated at 98 °C for 15 min. The samples were subsequently analyzed by western blot as described above.

To detect NE conjugation with ADRB3 in BAT by ELISA, BAT lysates were prepared by homogenizing 100 mg of tissue in 200 μL of PBS containing a protease and phosphatase inhibitor cocktail. The lysates were subsequently centrifuged at 12,000 rpm for 15 min to remove precipitates. A 20 μL aliquot of the lysate was mixed with 10 μL of 3× SDS-PAGE loading buffer to serve as the input sample. The remaining lysate was incubated with 5 μL of an anti-ADRB3 antibody (ABclonal, A8607) overnight at 4 °C. Protein A/G (Selleck, B23202) magnetic beads were blocked with 3% BSA to prevent nonspecific binding and then washed three times with PBS. The lysate-antibody mixture was added to the prepared beads and incubated at 4 °C for 4 h. The beads were subsequently washed three times with wash buffer and the protein was eluted with 50 μL of elution buffer at room temperature for 2 min. The supernatant was transferred to a new tube and neutralized immediately with 50 μL of 1 M Tris-HCl (pH 9.0). This neutralized sample was used for subsequent ELISA.

### CUT&Tag-seq and data analysis

CUT&Tag was performed with a Hyperactive Universal CUT&Tag Assay Kit for Illumina (Vazyme, TD903). C3H10T1/2 cells were harvested and counted after 6 h of treatment with 10 μM NE or vehicle. A total of 10,000 cells for each sample were washed with 500 μL of wash buffer and incubated with activated concanavalin A-coated (ConA) beads at room temperature for 10 min. After the supernatant was removed, the ConA beads were incubated with 100 μL of antibody buffer containing 2 μL of anti-phospho-CREB1 antibody at 4 °C overnight. After the supernatant was removed, the beads were incubated with goat anti-rabbit IgG (H + L) antibody (1:100) diluted in 100 μL of Dig wash buffer at room temperature for 1 h and then were washed with Dig wash buffer. Transposase pA/pG-Tn5 was used to induce DNA tagmentation, for which the beads were incubated with 0.04 μM pA/G-Tnp in Dig-300 buffer at room temperature for 1 h and then washed with Dig-300 buffer. The samples were incubated in Dig-300 Buffer with TTBL for 1 h at 37 °C for DNA fragmentation, followed by Proteinase K digestion and DNA extraction. The DNA extract beads provided with the kit were used for DNA extraction, followed by elution with 20 µL of DNase-free water. DNA was immediately subjected to PCR amplification by universal barcoded i5 and i7 primers from the TruePrep Index Kit V2 for Illumina (Vazyme, TD202). Given that 10,000 cells were used as input, the cycle number was set to 14 for PCR amplification. The amplified library was purified without size selection, and 100 μL of VAHTS DNA Clean Beads (Vazyme, N411) was added to the PCR product for incubation at room temperature for 5 min. The beads were then washed with wash buffer followed by elution with 22 μL of DNase-free water. Libraries were pooled to a final concentration of 5–10 nM and sequenced paired-end using the Illumina NovaSeq X Plus Series PE150 by Novogene. Data processing was carried out using R studio. The seqtk subsample was used to specify seed values to ensure that the extracted sequence pairs could maintain paired relationships. Trim Galore was used for quality control and low-quality data pruning of the sequencing data. The clean reads were then aligned to reference mm39 genome sequences by Bowtie2, which was subsequently used to compare the reads and produce SAM files. The dataset is displayed in the UCSC Genome Browser for visualization. Bamoverage was used to convert the BAM files to BigWig format.

### OCR measurement

The cellular OCR was measured using an oxygen meter with a Mitocell mixing chamber (Strathkelvin Instruments, MT200). Cells were suspended in 400 µL of cell culture medium to assess the basal OCR. Subsequently, 5 mg/mL oligomycin A (Selleck, S1478) and 5 μM FCCP (MCE, HY-100410) were added to measure the uncoupled and maximum OCRs, respectively. The OCRs were calculated using the 782 Oxygen System.

### ELISA

Human plasma sST2 levels were determined using a Human ST2 ELISA Kit (Proteintech, KE00055) according to the manufacturer’s instructions. Plasma samples were collected and diluted by 10-fold. sST2 levels in mouse plasma and culture medium were measured using a Quantikine ELISA Mouse ST2/IL-33 R Immunoassay Kit (R&D Systems, MST200) following the manufacturer’s instructions. Plasma samples were collected and diluted 10-fold, while medium samples were harvested from cells cultured in high-glucose DMEM for 24 h and added to the ELISA microplate without dilution. NE levels were measured using the NA/NE (Noradrenaline/Norepinephrine) ELISA Kit (Elabscience, E-EL-0047) according to the manufacturer’s instructions. Plasma samples and ADRB3 co-immunoprecipitation samples were collected as described above. For BAT and eWAT homogenates, tissues (5–20 mg for BAT and 10–50 mg for eWAT) were weighed, minced, and homogenized in 500 µL of PBS. The homogenates were then centrifuged at 10,000 rpm for 10 min at 4 °C to obtain the supernatant. Plasma and eWAT samples were diluted by 10-fold, BAT samples were diluted by 100-fold, and co-immunoprecipitated samples were used without dilution. The remaining steps followed the manufacturer’s protocol.

### Echocardiography

Mice were anesthetized with 3% isoflurane and maintained with 1% isoflurane mixed with 0.5 L/min oxygen. The anterior thoracic area was shaved to facilitate imaging. Echocardiographic analysis was performed using a High-Resolution Digital Imaging System (VisualSonics, Vevo 2100) equipped with a 25 MHz imaging transducer. To minimize confounding biases, heart rates were maintained between 450 and 500 bpm. M-mode ultrasound was conducted at the papillary muscle level to assess left ventricular function, including the left ventricular ejection fraction.

### Statistical analysis

Statistical analysis was performed using GraphPad Prism 10. Statistical differences were evaluated via two-tailed unpaired Student’s *t*-test for comparisons between two groups or analysis of variance (ANOVA) and appropriate post hoc analyses for comparisons of more than two groups. Analysis of covariance (ANCOVA) was used for statistical analysis of the VO_2_ and energy expenditure data, which was performed at https://calrapp.org. We used body weight as a covariate. *P* < 0.05 was considered statistically significant. The statistical methods and corresponding *P* values are included in the figure legends.

## Supplementary information

Supplementary information

## Data Availability

The RNA-seq and CUT&Tag-seq data are available in the Gene Expression Omnibus with accession numbers GSE297636 and GSE297635, respectively. All data used for the statistical analyses are available. Any additional information required to reanalyze the data reported in this paper is available from the corresponding author upon request.
