# Supplementary material for: WAT-to-BAT communication facilitates the sustained activation of BAT thermogenesis during cold exposure
Source: Cell Discov. 2026 May 26;12:37. doi: 10.1038/s41421-026-00891-8 (PMC13212969; doi:10.1038/s41421-026-00891-8)
Supplement: Supplementary file 1 — Supplementary information [file 41421_2026_891_MOESM1_ESM.pdf]

Supplemental Information

Figures

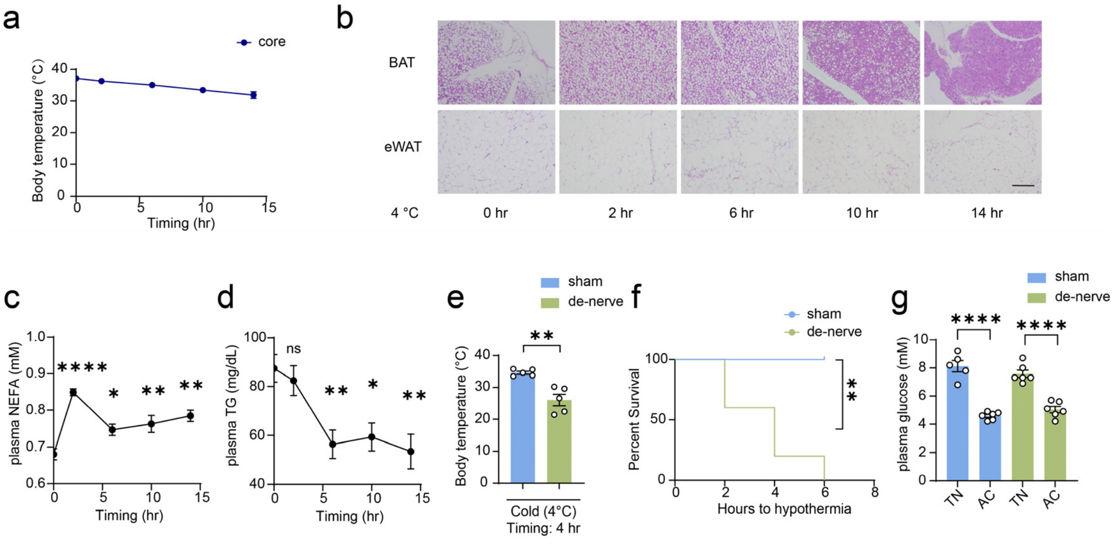

Figure S1

Supplementary Fig. S1 The progressive activation of BAT during cold exposure is mediated by integrated neural and humoral signals.

a, Line chart showing core body temperature of mice exposed to 4 °C at indicated time points (n=4 per group).

b, Hematoxylin and eosin (H&E) staining of BAT and eWAT sections from mice exposed to 4 °C at indicated time points. Scale bar: 100 μm.

c,**d**, Line charts showing plasma non-esterified fatty acid (NEFA, c) and triglyceride (TG, d) levels from mice exposed to 4 °C at indicated time points (n=4 per group).

e, Core body temperature of sham-operated or BAT-denervated mice exposed to 4 °C cold stimuli for 4 hours (n=5 per group).

f, Survival curves of sham-operated or BAT-denervated mice exposed to 4 °C cold stimuli for 6 hours. Mice were euthanized when their core temperature dropped below 30 °C (n=5 per group).

g, Glucose levels in plasma from sham-operated or BAT-denervated mice exposed to 30 °C (thermoneutrality, TN) or 10 °C (acute cold, AC) for 8 hours (n=5-6 per group).

Data are shown as mean ± SEM. Statistical significance was determined by two-tailed unpaired Student's t test in (e) and (g), Log-rank (Mantel-Cox) test in (f) and one-way ANOVA multiple comparison test in (c) and (d). * $p < 0.05$, ** $p < 0.01$, **** $p < 0.0001$, ns, not significant.

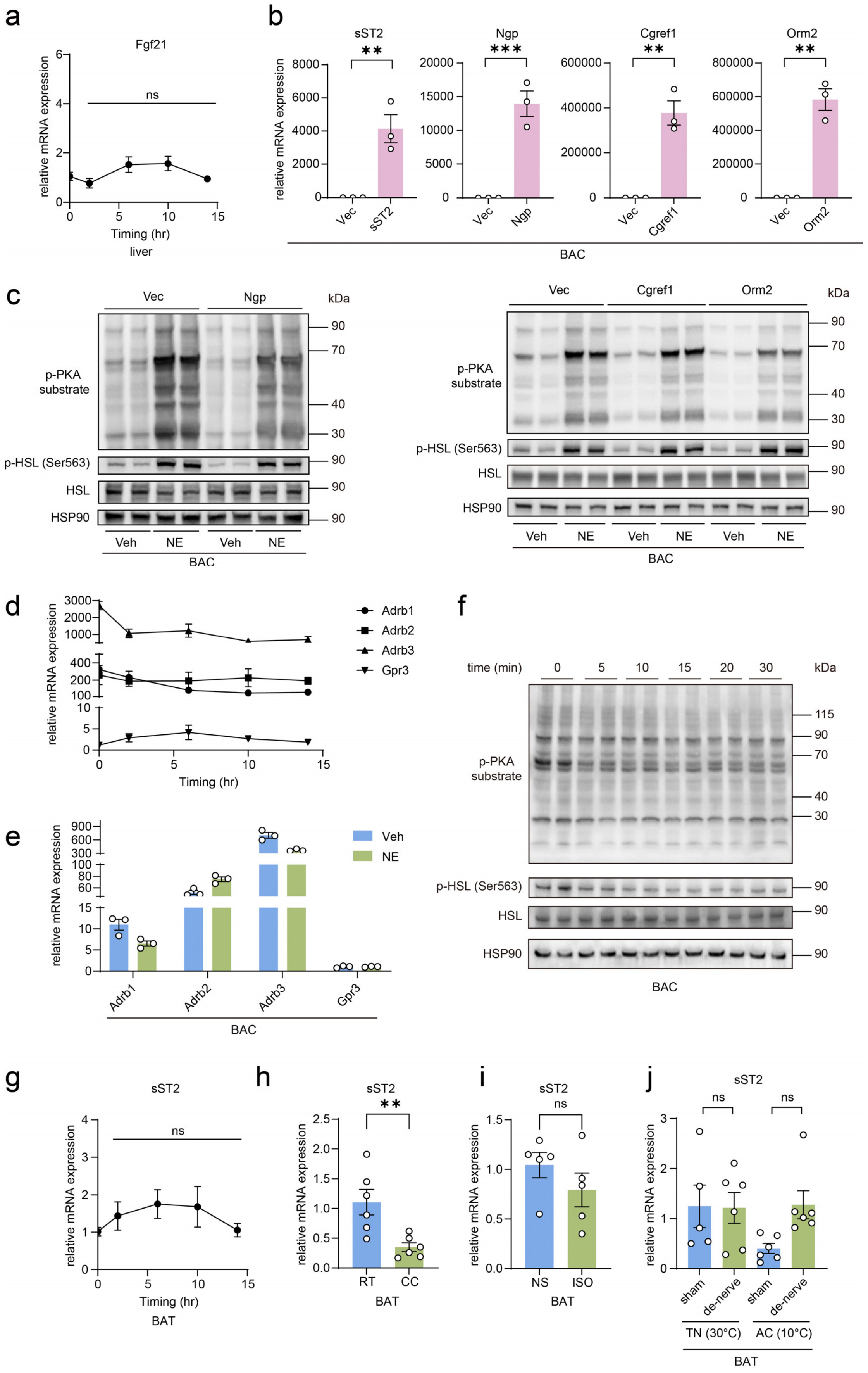
a
Fgf21
ns
relative mRNA expression
Timing (hr)
liver
b
sST2
Ngp
Cgref1
Orm2
Vec
BAC
c
Vec
Ngp
Cgref1
Orm2
kDa
p-PKA substrate
p-HSL (Ser563)
HSL
HSP90
Veh
NE
d
Adrb1
Adrb2
Adrb3
Gpr3
e
Veh
NE
f
time (min)
g
sST2
BAT
h
RT
CC
i
NS
ISO
j
sham
de-nerve
TN (30°C)
AC (10°C)

Figure S2

Supplementary Fig. S2 Identification of humoral mechanisms that mediates the activation of β-adrenergic receptor signaling in BAT

a, Line chart showing qPCR analysis of Fgf21 expression in the liver from mice exposed to 4 °C at indicated time points (n=4 per group).

b, QPCR analysis of humoral candidate gene expression in brown adipocytes (BAC) overexpressing vector (Vec), sST2, Ngp, Cgref1, or Orm2 (n=3 per group) after differentiation.

c, Immunoblotting showing the phosphorylation of PKA substrate and HSL in differentiated BAC overexpressing Vec and Ngp (left panel) or Vec, Cgref1 and Orm2 (right panel). Cells were treated with norepinephrine (NE, 1 μM) for 15 minutes prior to sample collection. HSP90 was used as a loading control.

d, Line chart showing qPCR analysis of the expression of Adrb1**,** Adrb2**,** Adrb3 and Gpr3 genes in BAT from mice exposed to 4 °C at indicated time points (n=4 per group).

e, QPCR analysis of the expression of Adrb1**,** Adrb2**,** Adrb3**,** and Gpr3 genes in differentiated BAC treated with vehicle (Veh) or NE (1 μM) for 6 hours (n=3 per group).

f, Immunoblotting showing the phosphorylation of PKA substrate and HSL in differentiated BAC treated with oleic acid (100μM) at indicated time points. HSP90 was used as a loading control.

g. Line chart showing qPCR analysis of sST2 expression in BAT from mice exposed to 4 °C at indicated time points (n=4 per group).

h. QPCR analysis of sST2 expression in BAT from mice (n=6 per group) treated with chronic cold exposure (CC) or room temperature (RT) for 7 days.

i. QPCR analysis of sST2 expression in BAT from mice (n=5 per group) intraperitoneally injected with saline (NS) or isoprenaline (ISO, 30 mg/kg/d) for 7 days.

j. QPCR analysis of sST2 expression in BAT from mice with sham operation or BAT denervation exposed to TN or AC (n=5–6 per group).

Data are shown as mean ± SEM. Statistical significance was determined by one-way ANOVA multiple comparison test in (a) and two-tailed unpaired Student's t test in (b) and (g-j). * $p < 0.05$, ** $p < 0.01$, ns, not significant.

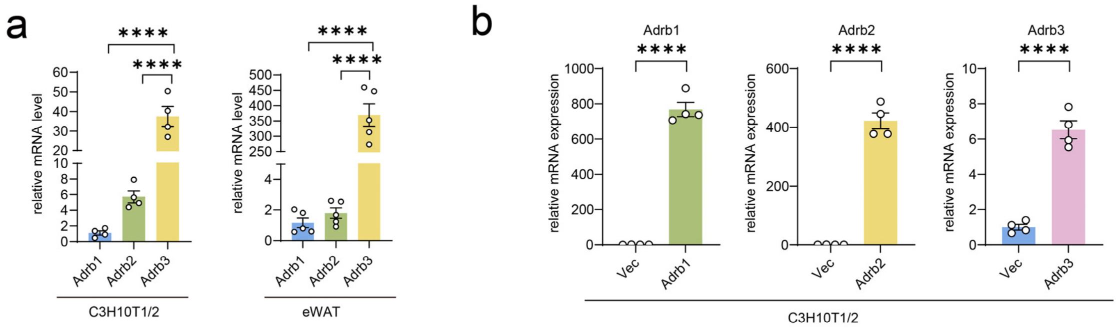

Figure S3

Supplementary Fig. S3 The expression of β-adrenoreceptors in white adipocytes

a, QPCR analysis of the expression of Adrb1, Adrb2 and Adrb3 in differentiated C3H10T1/2 cells (n=4) and eWAT from wild type mice (n=5).

b, QPCR analysis of the expression of Adrb1, Adrb2 and Adrb3 in C3H10T1/2 cells overexpressing vector (Vec), Adrb1, Adrb2 and Adrb3 after differentiation (n=4 per group). Data are shown as mean ± SEM. Statistical significance was tested by one-way ANOVA multiple comparison test in (a) and two-tailed unpaired Student's t test in (b). **** $p < 0.0001$.

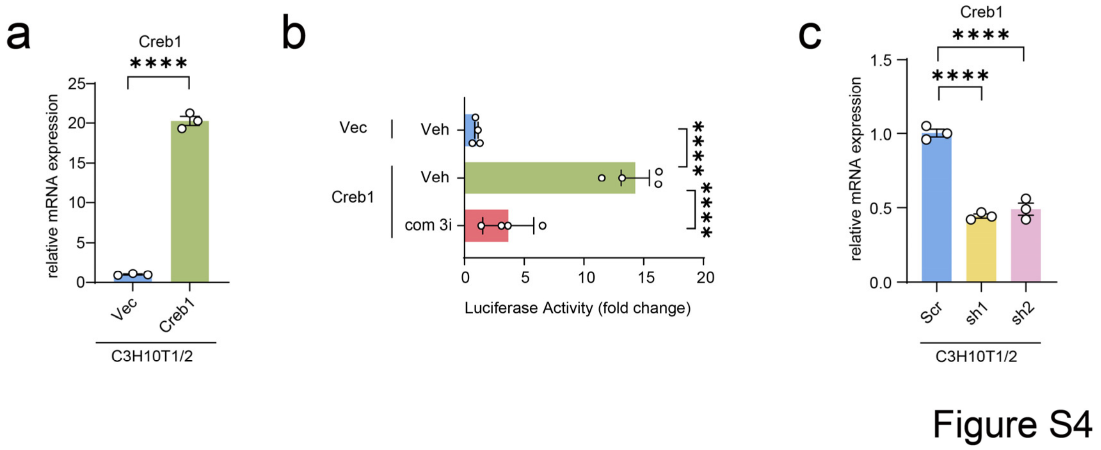

Supplementary Fig. S4 The transcription activity of Creb1 is inhibited by compound 3i

a, QPCR analysis of the expression of Creb1 in differentiated C3H10T1/2 cells expressing vector (Vec) or Creb1 (n=3 per group).

b, Luciferase activity in HEK293T cells cotransfected with the pGL3-UCP1 promoter-luciferase plasmid and vector (Vec) or pcDNA3-CREB1 (Creb1), followed by treatment with vehicle (Veh) or Compound 3i (com 3i) for 24 hours (n=3 per group).

c, QPCR analysis of Creb1 expression in C3H10T1/2 cells transfected with scramble (Scr) or two independent small hairpin RNAs targeting Creb1 (sh1 and sh2) after differentiation (n=3 per group).

Data are shown as mean ± SEM. Statistical significance was determined by two-tailed unpaired Student's t test in (a) and one-way ANOVA multiple comparison test in (b) and (c). **** $p < 0.0001$.

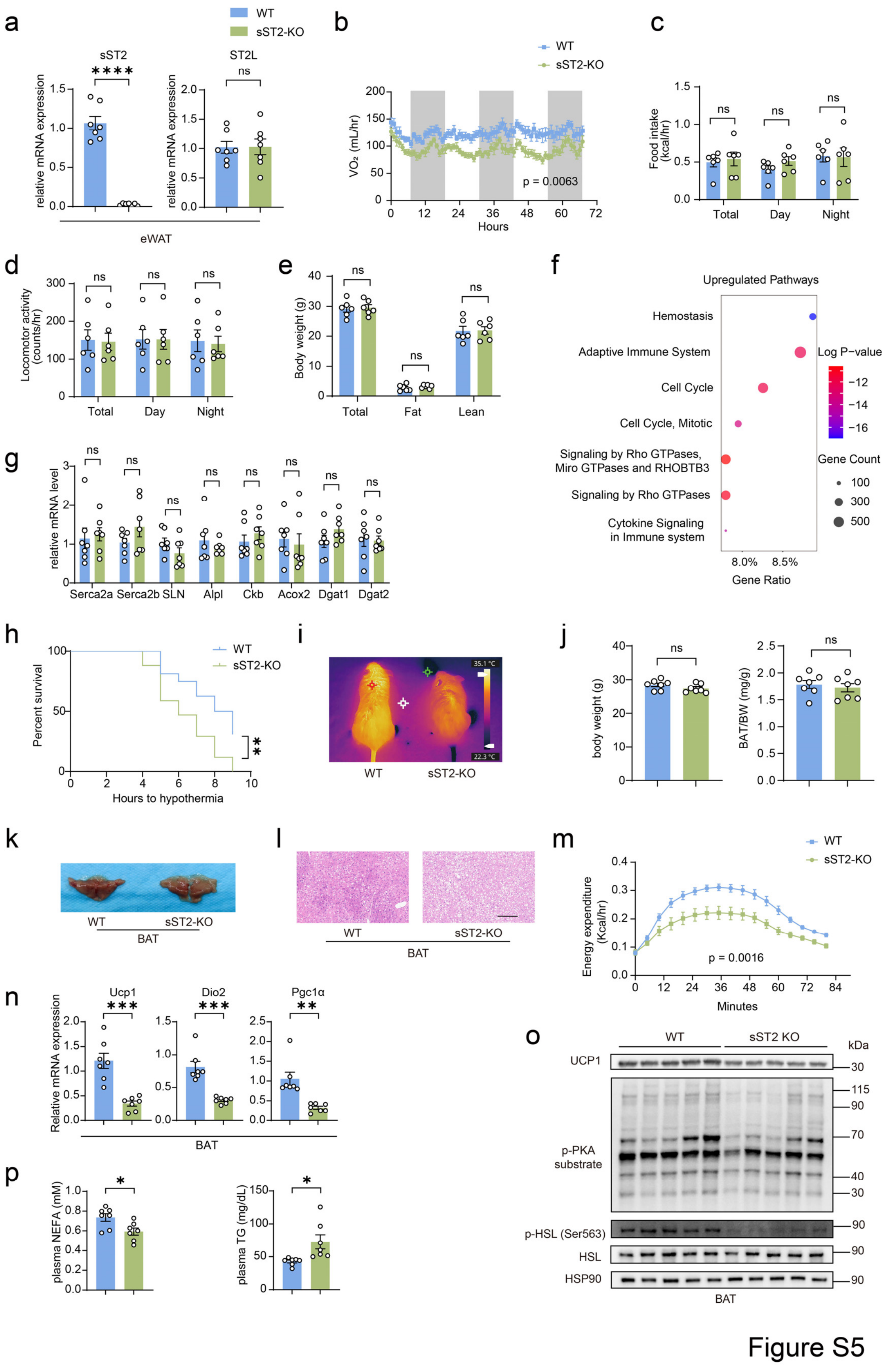

Figure S5

Supplementary Fig. S5 Depletion of sST2 impairs BAT thermogenesis in prolonged

cold exposure.

a, QPCR analysis of the expression of sST2 and ST2L gene in eWAT from wild type (WT) and sST2 KO mice (n=7 per group).

b,c,d, Line plots depict the oxygen consumption (b) recorded over a 68-hour period; and food intake (c) and locomotor activity (d) in WT and sST2 KO mice (n = 6 per group) following BAT denervation. All measurements were performed in metabolic cages under ambient room temperature conditions.

e, Total body weight, fat weight, and lean weight of WT and sST2 KO mice (n = 6 per group) following BAT denervation.

f, Bubble chart displaying the top 7 upregulated pathways in Reactome gene sets identified by Metascape enrichment analysis of upregulated differentially expressed genes in BAT from sST2 KO mice subjected to BAT denervation and exposed to 10 °C cold stimuli for 8 hours**.**

g, QPCR analysis of UCP1-independent thermogenic genes in BAT from WT and sST2 KO mice (n=7 per group) subjected to BAT denervation and exposed to 10 °C cold stimuli for 8 hours.

h, Survival curves of WT mice and sST2 KO mice exposed to 4 °C cold stimuli. Mice were euthanized when their core temperature dropped below 30 °C (n=16-17 per group).

i, Representative infrared thermography of WT and sST2 KO mice exposed to 4 °C cold stimuli for 6 hours.

j, Body weight and BAT weight to BW ratio of WT and sST2 KO mice exposed to 4 °C cold stimuli for 6 hours (n=7 per group).

k,l, Gross morphology (k) and H&E staining (l) of BAT from WT and sST2 KO mice exposed to 4 °C cold stimuli for 6 hours. Scale bar: 100 μm.

m, Energy expenditure of WT and sST2 KO mice (n = 7 per group) after NE (1 mg/kg) treatment.

n, QPCR analysis of thermogenic genes in BAT from WT mice and sST2 KO mice exposed to 4 °C cold stimuli for 6 hours (n=7 per group).

o, Immunoblotting showing the expression of UCP1, phosphorylated PKA substrate, phosphorylated HSL and total HSL in BAT from WT mice and sST2 KO mice exposed to 4 °C cold stimuli for 6 hours. HSP90 was used as a loading control.

p, Non-esterified fatty acid (NEFA) and triglyceride (TG) levels in plasma from WT mice and sST2 KO mice exposed to 4 °C cold stimuli for 6 hours (n=7 per group).

Data are shown as mean ± SEM. Statistical significance was determined by two-tailed unpaired Student's t test in (a), (c), (d), (e), (g), (j), (n) and (p), two-way ANOVA in (b) and (m) and Log-rank (Mantel-Cox) test in (h). * $p < 0.05$, ** $p < 0.01$, **** $p < 0.0001$, ns, not significant.

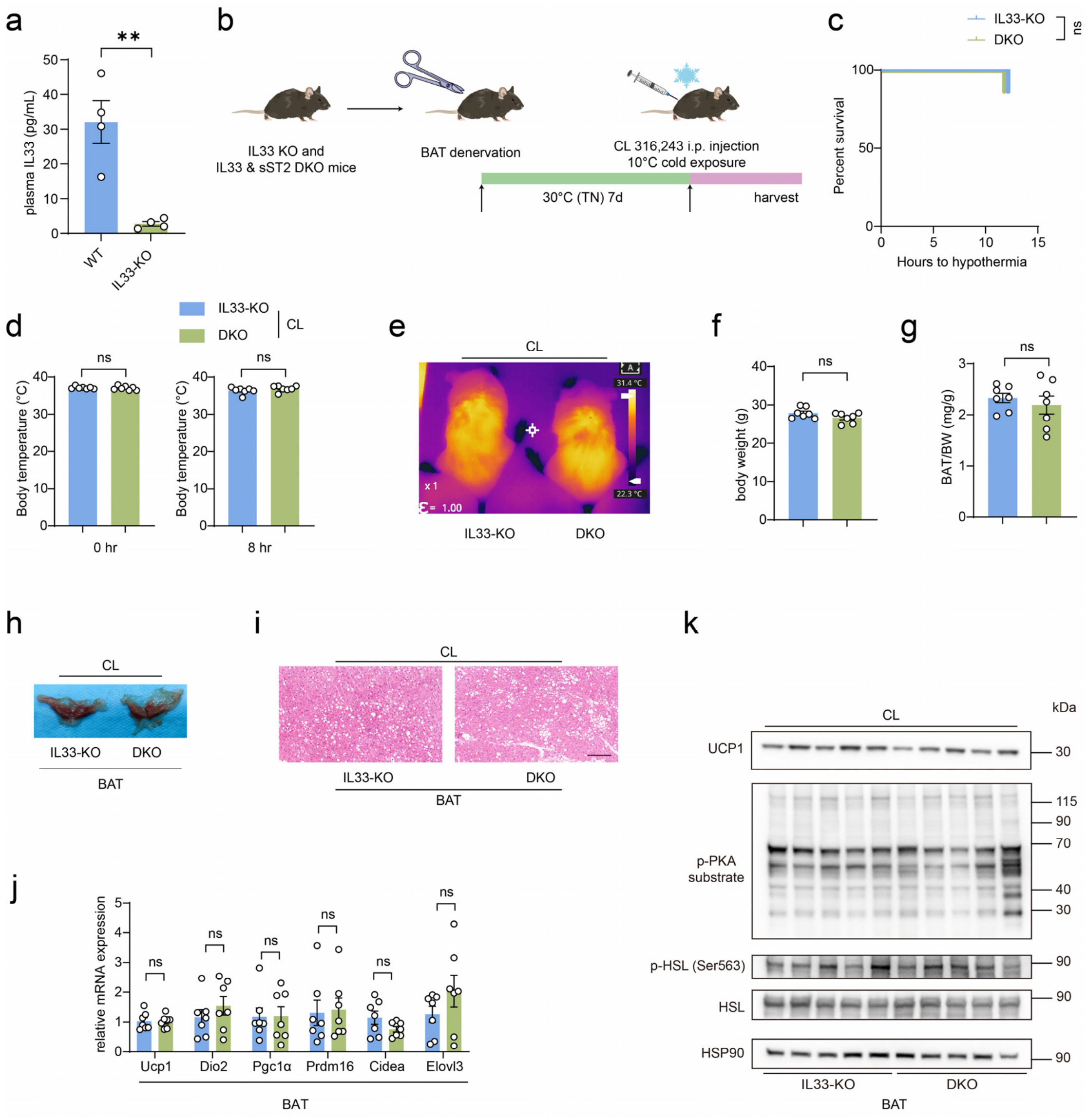

Figure S6

Supplementary Fig. S6 β3-adrenergic receptor agonist treatment restores BAT thermogenesis and alleviates cold intolerance in BAT-denervated sST2 knockout mice.

a, Plasma IL33 levels from 10-week-old wild type (WT) and Il33 knockout (Il33-KO) mice (n=4 per group).

b, Schematic depicting 10-week-old Il33 KO and sST2/Il33 double knockout (DKO) mice subjected to BAT denervation, followed by cold exposure at 10 °C and intraperitoneal administration of CL 316,243 (CL, 1 mg/kg) 30 minutes prior to cold challenge.

c,d, Survival curves (c) and core body temperature (d) of BAT-denervated IL33-KO and DKO mice after treatment with CL and subsequent exposure to 10 °C cold stimuli for 12 hours (n=7 per group).

e, Representative infrared thermography of BAT-denervated IL33-KO and DKO mice after

CL treatment and subsequent exposure to 10 °C cold stimuli for 8 hours.

f,g, Body weight (f) and BAT weight to BW ratio (g) of BAT-denervated IL33-KO mice and DKO mice after CL treatment and subsequent exposure to 10 °C cold stimuli for 8 hours (n=7 per group).

h,i, Gross morphology (h) and H&E staining (i) of BAT from BAT-denervated IL33-KO mice and DKO mice after CL treatment and subsequent exposure to 10 °C cold stimuli for 8 hours. Scale bar: 100 μm.

j, QPCR analysis of the expression of thermogenic genes in BAT from BAT-denervated IL33-KO mice and DKO mice after CL treatment and subsequent exposure to 10 °C cold stimuli for 8 hours (n=7 per group).

k, Immunoblotting showing the expression of UCP1, phosphorylated PKA substrate, phosphorylated HSL and total HSL in BAT from IL33 KO and DKO mice after CL treatment and subsequent exposure to 10 °C cold stimuli for 8 hours. HSP90 was used as a loading control.

Data are shown as mean ± SEM. Statistical significance was determined by two-tailed unpaired Student's t test in (a), (d), (f), (g) and (j); Log-rank (Mantel-Cox) test in (c). ** $p < 0.01$, ns, not significant.

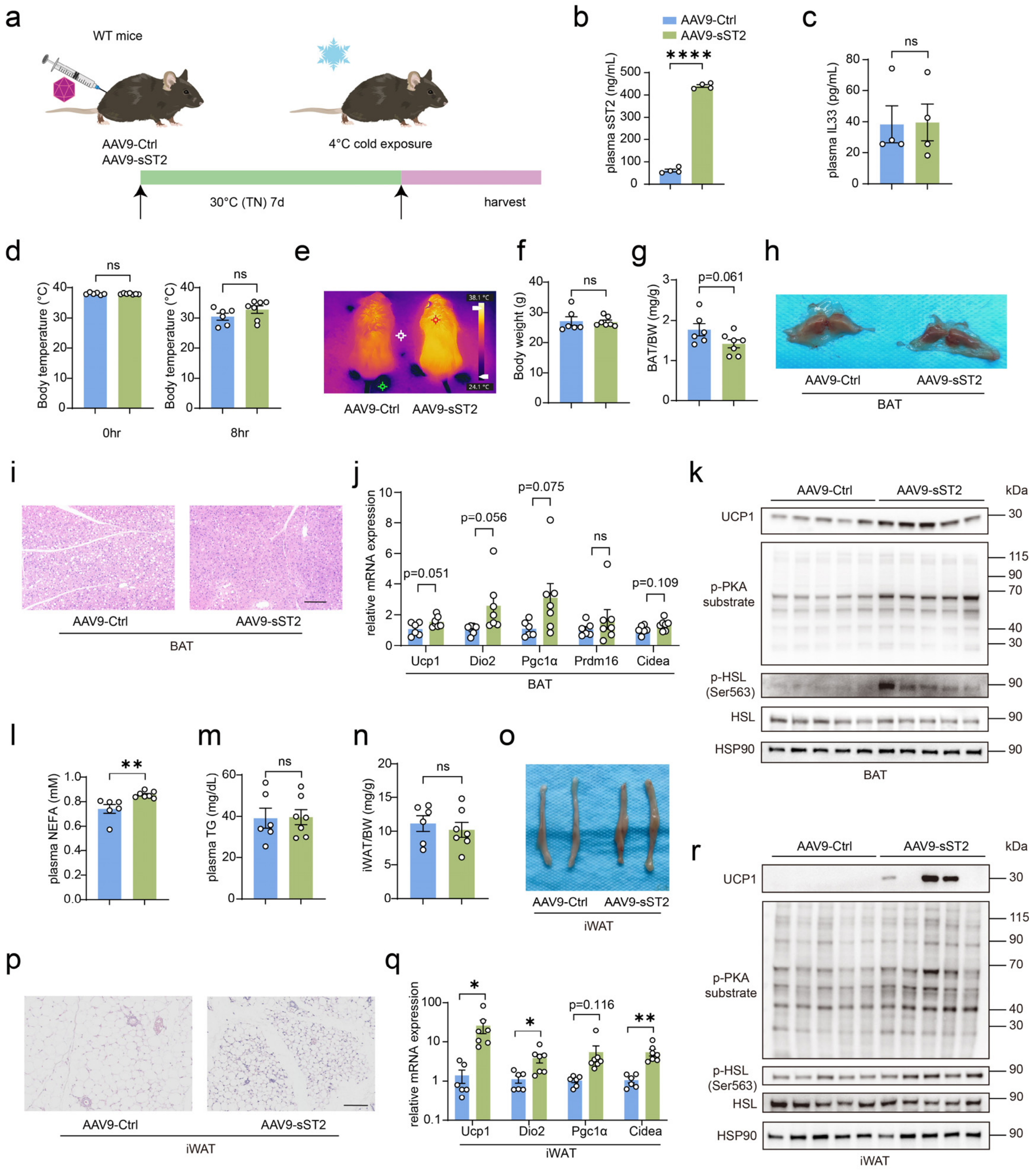

Figure S7

Supplementary Fig. S7 Overexpression of sST2 facilitates beige fat formation during cold exposure.

a, Schematic illustrating 10-week-old wild type (WT) mice subjected to tail vein injection of adeno-associated virus (AAV) followed by exposure to 4 °C cold stimuli for 8 hours**.**

b,c, Plasma sST2 (b) and IL33 (c) levels from WT mice after tail vein injection of AAV9-Ctrl or AAV9-sST2, followed by exposure to 4 °C cold stimuli for 8 hours (n=4 per group).

d, Core body temperature of WT mice injected with AAV9-Ctrl or AAV9-sST2 after 8 hours of cold exposure at 4 °C (n=6–7 per group).

e, Representative infrared thermography of WT mice injected with AAV9-Ctrl or AAV9-sST2 after 8 hours of cold exposure at 4 °C

f,g, Body weight (f) and BAT weight to BW ratio (g) of WT mice injected with AAV9-Ctrl or

AAV9-sST2 after 8 hours of cold exposure at 4 °C (n=6–7 per group)

h,i, Gross morphology (h) and H&E staining of BAT (i) from WT mice injected with AAV9-Ctrl or AAV9-sST2 after 8 hours of cold exposure at 4 °C. Scale bar: 100 μm.

j, QPCR analysis of thermogenic genes in BAT from WT mice injected with AAV9-Ctrl or AAV9-sST2 after 8 hours of cold exposure at 4 °C (n=6–7 per group).

k, Immunoblotting showing the expression of UCP1, phosphorylated PKA substrate, phosphorylated HSL and total HSL in BAT from WT mice injected with AAV9-Ctrl or AAV9-sST2 after 8 hours of cold exposure at 4 °C. HSP90 was used as a loading control.

l,m, Plasma non-esterified fatty acid (NEFA, l) and triglyceride (TG, m) levels from WT mice injected with AAV9-Ctrl or AAV9-sST2 after 8 hours of cold exposure at 4 °C (n=6–7 per group).

n, Inguinal white adipose tissue (iWAT) weight to BW ratio of WT mice injected with AAV9-Ctrl or AAV9-sST2 after 8 hours of cold exposure at 4 °C.

o,p, Gross morphology (o) and H&E staining (p) of iWAT from WT mice injected with AAV9-Ctrl or AAV9-sST2 after 8 hours of cold exposure at 4 °C. Scale bar: 100 μm.

q, QPCR analysis of thermogenic genes in iWAT from WT mice injected with AAV9-Ctrl or AAV9-sST2 after 8 hours of cold exposure at 4 °C (n=6–7 per group).

r, Immunoblotting showing the expression of UCP1, phosphorylated PKA substrate, phosphorylated HSL and total HSL in BAT from WT mice injected with AAV9-Ctrl or AAV9-sST2 after 8 hours of cold exposure at 4 °C. HSP90 was used as a loading control.

Data are shown as mean ± SEM. Statistical significance was determined by two-tailed unpaired Student's t test in (b-d), (f), (g), (j), (l-n) and (q), * $p < 0.05$, ** $p < 0.01$, **** $p < 0.0001$, ns, not significant.

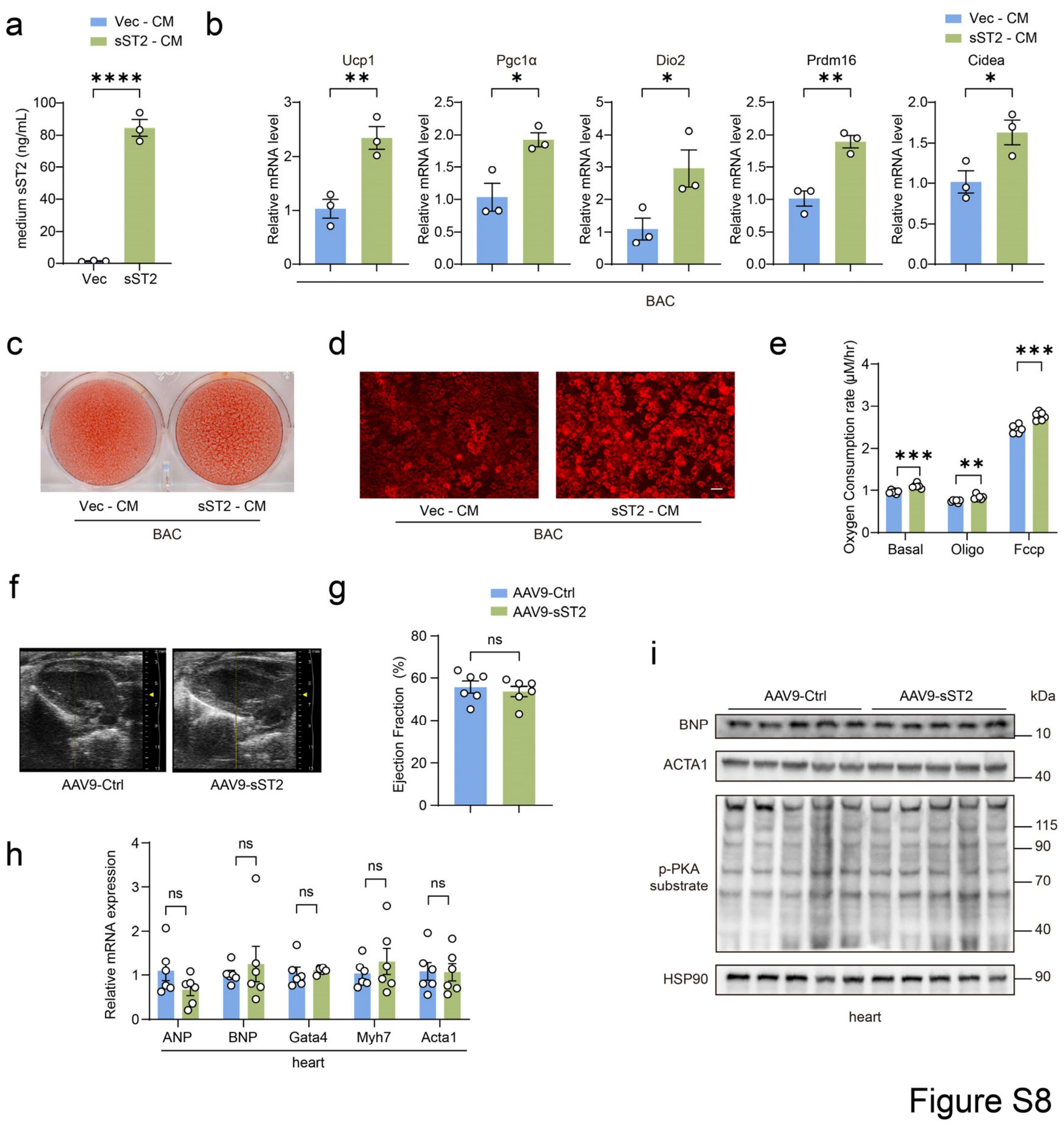

Supplementary Fig. S8 sST2 induces brown adipocyte differentiation, whereas it does not induce β-adrenergic activation in the heart or impair cardiac function.

a, Quantification of secreted sST2 levels in the culture medium of HEK293T cells following transfection with vector (Vec) or sST2-overexpressing plasmid (n=3 per group).

b, QPCR analysis of thermogenic genes expression in BAC treated with vector (Vec) and sST2 overexpressing conditional medium (CM) during differentiation (n=3 per group).

c,d, Oil red O (c) and MitoTracker staining (d) of BAC treated with Vec and sST2 CM during differentiation. Scale bar: 100 μm.

e, Oxygen consumption rate (OCR) of differentiated BAC treated with Vec and sST2 CM during differentiation. FCCP (10 μM) and oligomycin (Oligo, 10 μg/ml) was treated for evaluation of mitochondrial respiratory function (n=6 per group).

f,g, Representative echocardiography images (f) and left ventricular ejection fraction (EF) (g) of WT mice injected with AAV9-Ctrl or AAV9-sST2 after 8 hours of cold exposure at 4 °C (n=6 per group).

h, QPCR analysis of heart failure markers in heart from WT mice injected with AAV9-Ctrl or AAV9-sST2 after 8 hours of cold exposure at 4 °C (n=6 per group).

i, Immunoblotting of heart failure markers and phosphorylation of PKA substrate in heart from WT mice injected with AAV9-Ctrl or AAV9-sST2 after 8 hours of cold exposure at 4 °C. HSP90 was used as a loading control.

Data are shown as mean ± SEM. Statistical significance was determined by two-tailed unpaired Student's t test in (a), (b), (e), (g) and (h). * $p < 0.05$, ** $p < 0.01$, *** $p < 0.001$, **** $p < 0.0001$, ns, not significant.

Table S1. List of shRNA sequences.

Creb1	
sh1	GACTGATGGACAGCAGATTCTA
sh2	GAAGAGAGAGGTCCGTCTAAT

Table S2. List of qPCR primers

Gene	Former	Reverse
mouse qPCR primers		
Ucp1	GGCATTCAGAGGCAAATCAGCT	CAATGAACACTGCCACACCTC
Elovl3	TTCTCACGCGGGTTAAAAATGG	GAGCAACAGATAGACGACCAC
Dio2	GATGCTCCCAATTCCAGTGT	TGAACCAAAGTTGACCACCA
Pgc1α	AGCCGTGACCACTGACAACGAG	GCTGCATGGTTCTGAGTGCTAAG
sST2	AGGCTGAGAAGGAAACAACCAA	TGCAGTGCACAGCTGATTCA
Prdm16	CGGAAGAGCGTGAGTACAAATG	TCCGTGAACACCTTGACACAGT
Cidea	GCAGCCTGCAGGAACTTATCAGC	GATCATGAAATGCGTGTTGTCC
Ebf2	GGAACCGGAACGAGACCCCT	TCCCTTGGGTTTCCCGCTGT
PPARα	GCAGTGCCCTGAACATCGA	CGCCGAAAGAAGCCCTTAC
Plin1	CACCATCTCTACCCGCCTTC	AGGGGCTGACTCCTTGTCTG
ANP	GCTTCCAGGCCATATTGGAG	GGGGGCATGACCTCATCTT
BNP	GAGGTCACTCCTATCCTCTGG	GCCATTTCCTCCGACTTTTCTC
Gata4	CCCTACCCAGCCTACATGG	ACATATCGAGATTGGGGTGTCT
Myh7	ACTGTCAACACTAAGAGGGTCA	TTGGATGATTTGATCTTCCAGGG
Acta1	CCCAAAGCTAACCGGGAGAAG	CCAGAATCCAACACGATGCC
Fgf21	CTGGGGGTCTACCAAGCATA	CACCCAGGATTTGAATGACC
Ngp	TCTAGTCAGAATCCTGCTACCAA	AGGAAGTCGCAGTCTTTAGGC
Adrb1	CTCATCGTGGTGGGTAACGTG	ACACACAGCACATCTACCGAA
Adrb2	GGGAACGACAGCGACTTCTT	GCCAGGACGATAACCGACAT
Adrb3	GGCCCTCTCTAGTTCCCAG	TAGCCATCAAACCTGTTGAGC
Gpr3	CCTGCTGGTGGGTAGCTTG	CATCACATAAGTCCGAGTTACCG
Creb1	AGCCGGGTACTACCATTCTAC	GCAGCTTGAACAACAACTTGG
ST2L	TGCATTTATGGGAGAGACCTGTTA	TGTGCAGAGCAATCTCCTGC
human qPCR primers		
Ucp1	GCAGGGAAAGAAACAGCACC	CTTTCACGACCTCTGTGGGT
sST2	GAAGTGGAAATTGGAAAAAACGC	GAAAGTTCAGGTGATCAAAGTCTCAG
ST2L	AAAATGTTCTGGATTGAGGCCAC	CCACTGCAGTGACTACATCTTCTCC
Cgref1	ACGATGACAGTGTTAATCCTGC	CCTAGTCCCTTTAGGTAGCTCTG
Orm2	GCTGTTCCTTAGGGACACCAA	TGACATCTGACCTGGGAATGC

Table S3. List of primary antibodies

Antibodies	Source	Catalog Number
ADRB1	Proteintech	28323-1-AP
ADRB2	Proteintech	29864-1-AP
ADRB3	Abclonal	A8607
UCP1	4ADI	UCP11-A
p-PKA substrate	CST	#9624
HSL	CST	#4107
p-HSL (Ser563)	CST	#4139
CREB	CST	#9197
p-CREB (Ser133)	CST	#9198
HSP90	Proteintech	13171-1-AP
DYKDDDK FLAG	Proteintech	66008-4-Ig
HA	Proteintech	51064-2-AP
ACTA1	Proteintech	23660-1-AP
BNP	Invitrogen	PA5-96084
